# Diabetes Mellitus Secondary to Endocrine Diseases: An Update of Diagnostic and Treatment Particularities

**DOI:** 10.3390/ijms241612676

**Published:** 2023-08-11

**Authors:** Mihaela Simona Popoviciu, Lorena Paduraru, Raluca Marinela Nutas, Alexandra Maria Ujoc, Galal Yahya, Kamel Metwally, Simona Cavalu

**Affiliations:** 1 Faculty of Medicine and Pharmacy, University of Oradea, P-ta 1 Decembrie 10, 410073 Oradea, Romania; elapopoviciu@yahoo.com (M.S.P.); lorenapaduraru93@yahoo.ro (L.P.); simona.cavalu@gmail.com (S.C.); 2 Bihor County Emergency Clinic Hospital, 410167 Oradea, Romania; popa_raluca_marinela@yahoo.com (R.M.N.); alexandraujoc@yahoo.com (A.M.U.); 3 Department of Microbiology and Immunology, Faculty of Pharmacy, Zagazig University, Zagazig 44519, Egypt; 4 Department of Medicinal Chemistry, Faculty of Pharmacy, University of Tabuk, Tabuk 71491, Saudi Arabia; kametwally@ut.edu.sa; 5 Department of Pharmaceutical Medicinal Chemistry, Faculty of Pharmacy, Zagazig University, Zagazig 44519, Egypt

**Keywords:** diabetes, acromegaly, endocrine diseases, Cushing’s syndrome, pheochromocytoma, Graves’ disease, aldosteronism, somatostatinoma, glucagonoma

## Abstract

Secondary diabetes mellitus is frequently ignored in specialized literature. In this narrative review, the main endocrinopathies accompanied by increased glycemic values are identified, as well as the mechanisms by which the excess or deficiency of certain hormones impact beta cell function or insulin resistance. The main endocrinopathies (acromegaly, Cushing’s syndrome, Basedow–Graves’ disease, pheochromocytoma, somatostatinoma and glucagonoma) and their characteristics are described along with the impact of hormone changes on blood sugar, body mass index and other parameters associated with diabetes. The overall information regarding the complex molecular mechanisms that cause the risk of secondary diabetes and metabolic syndrome is of crucial importance in order to prevent the development of the disease and its complications and particularly to reduce the cardiovascular risk of these patients. The purpose of this study is to highlight the particular features of endocrine pathologies accompanied by an increased risk of developing diabetes, in the context of personalized therapeutic decision making. The epidemiological, physiopathological, clinical and therapeutic approaches are presented along with the importance of screening for diabetes in endocrine diseases.

## 1. Introduction

Diabetes mellitus is a chronic disease, with a marked impact on quality of life, considered one of the most important and common diseases encountered in the medical clinic and which at the present time, due to the multiple complications and comorbidities involved in this pathology, represents a real risk at the global level [1]. Diabetes and prediabetes frequently appear as manifestations of well-known endocrine diseases. Moreover, the presence of diabetes represents an additional risk factor for cardiovascular mortality, but also a factor that influences and personalizes therapeutic decision making. Usually, diabetes secondary to endocrine diseases can be cured when the hormonal excess is corrected [2].

Diabetes caused by an endocrine disorder can be falsely considered type 2 diabetes. The ADA classification of diabetes includes four categories: type 1 diabetes characterized by the destruction of pancreatic β cells and absolute insulin deficiency, type 2 diabetes characterized by the progressive deficiency of insulin secretion on the background of insulin resistance, gestational diabetes and other specific types of diabetes due to other causes (for example, genetic defects of pancreatic β cell function or insulin action, diseases of the exocrine pancreas, endocrinopathies, drug-induced diabetes or diabetes caused by chemicals, diabetes caused by infections such as congenital rubella, cytomegalovirus) [3]. The criteria for the diagnosis of diabetes are a fasting plasma glucose ≥ 126 mg/dL (7 mmol/L) or HbA1c ≥ 6.5% or plasma glucose at 2 h ≥ 200 mg/dL (11.1 mmol/L) during an oral glucose tolerance test or plasma glucose at any time of the day ≥ 200 mg/dL (11.1 mmol/L) in a patient with diabetes symptoms. The International Expert Committee reports on the role of the A1C assay in the diagnosis of diabetes [4].

Prediabetes is an intermediate stage between normal glucose tolerance and diabetes and includes altered basal blood glucose and altered glucose tolerance. The criteria that define prediabetes are fasting plasma glucose values between 100 and 125 mg/dL (5.6–6.9 mmol/L) for modified basal glucose or plasma glucose 2 h after oral loading with 75 g of glucose between 140 and 199 mg/dL (7.8–11 mmol/L) for the alteration of glucose tolerance or HbA1c between 5.7 and 6.4% [5].

According to the International Diabetes Federation (IDF) Atlas, in 2021, approximately 537 million people had diabetes mellitus, and this number is estimated to reach 643 million by 2030 and 783 million by 2045. In 2021, approximatively 6.7 million people, in the age group 20–79, died from diabetes mellitus. The increased prevalence of diabetes worldwide is one of the most worrying aspects of this pathology. Considering these data, it is a necessity to study this pathology, including the diseases that might lead to its occurrence [6].

This paper reviews the epidemiological, physiopathological, clinical and therapeutic aspects of diabetes in patients with acromegaly, Cushing’s syndrome, pheochromocytoma, primary hyperaldosteronism, Basedow–Graves’ disease, somatostatinoma and glucagonoma, as the main identified endocrinopathies.

## 2. Diabetes Mellitus Secondary to Acromegaly

Acromegaly is a clinical syndrome caused by the excessive secretion of growth hormone. It is a multisystem disease characterized by excessive somatic growth, multiple comorbidities, premature mortality and dysmorphism. The excessive secretion of growth hormone that occurs before the closure of the growth cartilage in a child or adolescent is called pituitary gigantism [7].

In most cases, acromegaly is the result of a growth-hormone-secreting pituitary adenoma. Other causes of acromegaly are multiple endocrine neoplasia-1, McCune–Albright syndrome, ectopic pituitary adenoma with a sphenoidal location or in the parapharyngeal sinuses, iatrogenic excess of growth hormone, excessive secretion of central growth-hormone-releasing hormone (GHRH) (hamartoma, choristoma or ganglioneuroma) and peripheral (small-cell lung cancer, medullary thyroid carcinoma, pheochromocytoma, adrenal adenoma, insulinoma) [8].

Growth hormone (GH) is secreted at the level of the adenohypophysis by somatotropic cells, the maximum levels being reached at night, at the onset of sleep. Its secretion is characterized by minimal basal secretion and episodes of pulsatile secretion. GHRH is a hypothalamic peptide that stimulates GH synthesis and release, while somatostatin has the role of inhibiting GH secretion. GHRH is secreted in discrete pulses that induce pulsatile GH secretion, and somatostatin controls the basal GH level. GH binds to the GH receptor predominantly located in the liver, and the receptor–ligand coupling favors the intracellular synthesis and secretion of IGF-1 (insulin-like growth factor-1). Circulating IGF-1 then binds to the IGF-1 receptor in peripheral tissues [9].

Unlike GH, the serum levels of IGF-1 are constant over a 24 h period. There are numerous factors involved in the regulation of growth hormone secretion: stress, physical exercise, nutritional status (GH secretion is lower in people with obesity), hormones (estrogen stimulates GH secretion, excess glucocorticoids inhibit GH release). Also, IGF-1 inhibits GH secretion through negative feedback [10].

The diagnosis of acromegaly must be taken into account in patients who present an increase in the size of the hands and feet, tightening of facial features with the growth of the mandible and prognathism, frontal bumps, growth of the nasal pyramid, macroglossia, dental diastemas followed by premature teething, excessive sweating, voice thickness, thickened integuments, arthropathy, kyphosis, carpal tunnel syndrome, sleep apnea and oligomenorrhea. Acromegaly is accompanied by an increased risk of developing diabetes, the appearance of colonic polyps and colon cancer, but also of cardiac conditions, such as hypertension, left ventricular hypertrophy and ischemic heart disease. The symptoms due to the direct compressive effects of the tumor mass are headache, visual field defects and paralysis of the cranial nerves. Serum IGF-1 dosage is an effective screening method when clinical manifestations raise the suspicion of acromegaly. Due to the pulsatile secretion of GH, a single determination has no absolute value for confirming the diagnosis [11,12]. To establish the diagnosis of acromegaly, the oral glucose tolerance test is used. Serum GH levels measured every 30 min for 2 h after glucose administration fall below 1 µg/L in most healthy individuals, whereas patients with acromegaly have a GH value above 1 µg/L. Once the diagnosis has been confirmed, a pituitary MRI must be performed to confirm the presence of the pituitary adenoma [13].

Acromegaly is frequently accompanied by metabolic comorbidities, mainly by disorders of carbohydrate and lipid metabolism, representing additional risk factors for increased cardiovascular mortality. Disorders of carbohydrate metabolism include altered basal blood glucose, altered glucose tolerance and diabetes. The prevalence of these disorders of carbohydrate metabolism varies considerably between different studies. The prevalence of altered basal glucose varies between 7 and 22%, of altered glucose tolerance between 6 and 45% and of diabetes between 19 and 56% [14]. A study that included 148 patients diagnosed with acromegaly between 1990 and 2010 showed that more than 50% of patients with acromegaly had an altered glycemic status at the time of diagnosis, 28.5% of patients with diabetes, 26.5% with prediabetes. Also, the study showed that, just like in the general population, diabetes and prediabetes in acromegaly are correlated with a family history of diabetes, older age and a higher body mass index [15]. Another study conducted by Ciresi and colleagues in which 157 men and 150 women newly diagnosed with acromegaly were included demonstrated that most of the metabolic characteristics in acromegaly are more strongly associated with the female sex, with women exhibiting more severe insulin resistance compared to men [16]. The main disorders of lipid metabolism include a decrease in HDL-cholesterol values, with a prevalence between 33 and 40%, and hypertriglyceridemia, whose prevalence varies between 39 and 47% [14].

Physiologically, GH exerts its effects both directly by inducing gluconeogenesis, glycogenolysis and lipolysis and promoting insulin resistance in the liver and peripheral tissues, as well as indirectly through IGF-1, which facilitates the action of insulin [8]. The pathogenesis of insulin resistance in acromegaly is due to multiple factors. GH exerts a lipolytic effect by determining the hydrolysis of triglycerides and the production of free fatty acids from adipose tissue, and this increased synthesis of free fatty acids leads to a decrease in insulin-mediated glucose uptake by inhibiting glucose transporters in adipose tissue [17]. Moreover, GH suppresses key insulin signaling pathways involved in stimulating glucose transport in muscle and adipose tissue and in inhibiting glucose production in the liver [18].

The resistance to insulin secondary to the excess of growth hormone is compensated by the increased secretion of insulin from the pancreatic β cells, and over time, with the decrease in the secretory capacity of the pancreas, prediabetes and diabetes can set in. Therefore, the impairment of pancreatic β cell function with the subsequent reduction in insulin secretion contributes significantly to the occurrence of glucose metabolism disorders [19]. Once the β cell function is affected, the glucose metabolism disorders persist even after the acromegaly is cured [20]. Although physiologically IGF-1 improves glucose homeostasis, the chronic excess of GH in acromegaly that causes insulin resistance greatly exceeds the possible beneficial effects of IGF-1 on insulin sensitivity [21].

The first treatment option for most patients with acromegaly is a transsphenoidal surgical resection of the GH-secreting adenoma. Following surgical excision, both insulin sensitivity and insulin secretion improves, and in 23–58% of patients with pre-existing diabetes, glucose metabolism returns to normal [20]. Drug therapy and radiotherapy are generally second-line and third-line treatment options, having indications of being preoperative adjuvant treatments to reduce the size of large macroadenomas, in patients with multiple comorbidities, in patients who refuse surgical intervention or in the case of postoperative recurrences [8]. Generation I somatostatin analogs (octreotide and lanreotide) exert their therapeutic effect mainly through the somatostatin receptor 2 (SSTR2) and, to a lesser extent, through the SSTR5 receptor, causing a decrease in GH secretion and a decrease in secretory tumor formation [22]. Somatostatin analogs of the first generation reduce insulin resistance but also the pancreatic secretion of insulin and glucagon. Thus, the progressive improvement in insulin resistance can counterbalance the inhibitory effect on insulin secretion, which appears mainly at the initiation of treatment, and in the long term, glucose tolerance can be improved [23]. Although some studies have shown an impairment of glycemic control, others have not confirmed these results, and the effect of first-generation somatostatin analogs on glucose metabolism remains controversial [24].

Pasireotide is a second-generation somatostatin analog with an increased affinity for the SSTR5 receptor and has been shown to be superior to octreotide or lanreotide in the treatment of acromegaly. It is recommended in cases where surgical intervention is ineffective or cannot be performed, as well as after the failure of therapy with first-generation somatostatin analogs. Pasireotide has an increased frequency of hyperglycemia and diabetes [24].

The PAOLA study carried out over a period of 6 months compared pasireotide with octreotide and lanreotide autogel. Diabetes occurred in 21% of patients treated with pasireotide LAR 40 mg and 26% of patients treated with pasireotide LAR 60 mg, compared to 8% of patients treated with octreotide and lanreotide [25].

The increased affinity for the SSTR5 receptor can be used to explain the hyperglycemic effects of pasireotide. Pancreatic α cells that secrete glucagon predominantly express the SSTR2 receptor, and β cells mainly express SSTR2 and SSTR5 receptors. Following binding to SSTR5, pasireotide suppresses insulin secretion, while having a modest inhibitory effect on glucagon secretion. This glucagon–insulin imbalance could explain the occurrence of hyperglycemia. In addition, binding to SSTR5 inhibits GLP-1 (glucagon-like peptide-1) secretion [24].

Patients who develop diabetes after treatment with pasireotide require the initiation of antidiabetic therapy, the first line being metformin. Dipeptidyl peptidase 4 inhibitors and GLP-1 receptor agonists can be effective in reducing hyperglycemia, being considered in stage II of the treatment [26].

Pegvisomant antagonizes the action of GH by peripherally blocking the binding of GH to its receptor and the consequent decrease in the level of IGF-1. It is indicated in stage II treatment of acromegaly, for patients with resistance or intolerance to somatostatin analogs. Clinical studies have shown that pegvisomant normalizes serum IGF-1 levels in over 90% of patients. Regarding the effect on carbohydrate metabolism, multiple studies have shown that pegvisomant reduces fasting glucose and HbA1c levels and improves glucose tolerance, even in patients with diabetes or impaired glucose tolerance [21]. It is not clear whether the positive effect of pegvisomant on glycemic disorders is due to the decrease in IGF-1 value or its specific action on glycemia [27].

Cabergoline and bromocriptine are dopaminergic agonists that modestly inhibit GH secretion, being used especially in cases with simultaneous GH and prolactin secretion, in monotherapy or more frequently in combination with somatostatin analogs. Cabergoline is more effective than bromocriptine. There are few data on the impact of dopaminergic agonists on glycemic control in acromegaly [24]. In the case of patients with type 2 diabetes without acromegaly, it has been proven that bromocriptine reduces the level of HbA1c and fasting blood sugar [28].

If antidiabetic therapy is necessary, diabetes should be managed similarly to the general population, with metformin being considered the first-line medication. Adverse effects of metformin such as diarrhea and abdominal pain have a greater significance for patients under treatment with somatostatin analogs which are known to cause similar adverse reactions. Diet change and weight loss are less important in the case of patients with acromegaly because they generally do not have visceral obesity, and insulin resistance is influenced by excess GH and IGF-1 [29].

**Figure 1:**
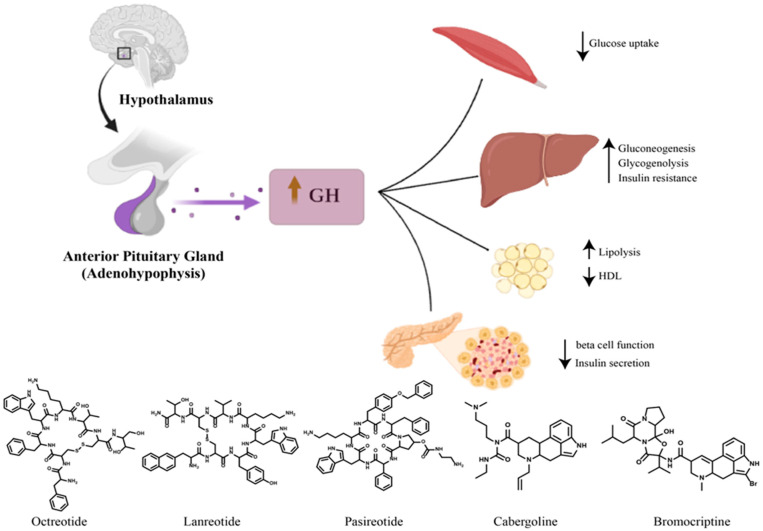
Diabetes secondary to acromegaly: diabetogenic effects of excessive growth hormone in the case of acromegaly and the available therapeutics for acromegaly. Hypothalamus (outlined with a black box) up arrows indicate increase or excess, while down arrows indicate decrease. (Figure originally created by the authors using BioRender: Scientific Image and Illustration Software https://www.biorender.com/).

GLP-1 receptor agonists and dipeptidyl peptidase 4 inhibitors should be considered as second-line treatments in individual cases, having an important role in the treatment of pasireotide-induced hyperglycemia. Diabetic patients with acromegaly could also be treated with SGLT-2 (sodium–glucose transport protein 2) inhibitors, although caution is required for patients with an uncontrolled disease. Insulin therapy should be considered especially when pancreatic β cell function is affected by a long-term hypersecretion of GH [21,30,31]. Figure 1 summarize the diabetogenic effects of excessive growth hormone in the case of acromegaly and the available therapeutics for acromegaly.

## 3. Cushing’s Syndrome and Diabetes

Cushing’s syndrome is characterized by a chronic excess of glucocorticoids, regardless of etiology. Cushing’s syndrome can be ACTH-dependent or ACTH-independent, caused by an ACTH-secreting pituitary adenoma (Cushing’s disease) or by the ectopic secretion of ACTH determined by lung, pancreatic or thymic carcinoid tumors, respectively, by a secreting adrenal cortical adenoma or carcinoma of cortisol, iatrogenic (through corticotherapy), ACTH-independent bilateral macronodular adrenal hyperplasia and primary pigmented nodular adrenal disease. Cushing’s disease is the most common form of endogenous Cushing’s syndrome, representing 80% of cases [32,33].

Cortisol is a steroid hormone produced in the fasciculated area of the adrenal glands. Its production is under the control of the hypothalamic–pituitary–adrenal cortical axis. ACTH is the main factor that stimulates cortisol secretion [34]. Stress, pain, physical exertion, trauma, infections and hypoglycemia activate the hypothalamic–pituitary–adrenal cortical axis [34,35].

The first step in the diagnosis of Cushing’s syndrome is the exclusion of the exogenous administration of glucocorticoids. Free urinary cortisol determined in urine/24 h reflects the concentration of free plasma cortisol, being a good screening test for Cushing’s syndrome [36]. The measurement of nocturnal salivary cortisol is another screening test, saliva being collected between 11 and 12 p.m. [37]. The nocturnal inhibition test with dexamethasone consists in the administration of 1 mg of dexamethasone between 23 and 24 h and a measurement of serum cortisol the following morning [38]. The inhibition test with dexamethasone for 48 h, 2 mg/day, is a confirmatory test [36].

The next stage of diagnosis consists in performing tests to establish the etiopathogenic form of Cushing’s syndrome by measuring the plasma ACTH that differentiates the ACTH-dependent and ACTH-independent forms of Cushing’s [39]. To distinguish between Cushing’s disease and ectopic ACTH secretion, an inhibition test with high-dose dexamethasone or a stimulation test with CRH or desmopressin can be used [40,41].

Disorders of carbohydrate metabolism occur in 14–74% of patients with Cushing’s syndrome. The prevalence of diabetes varies between 21 and 47%, and of modified basal blood glucose, between 21 and 64% [42].

**Figure 2:**
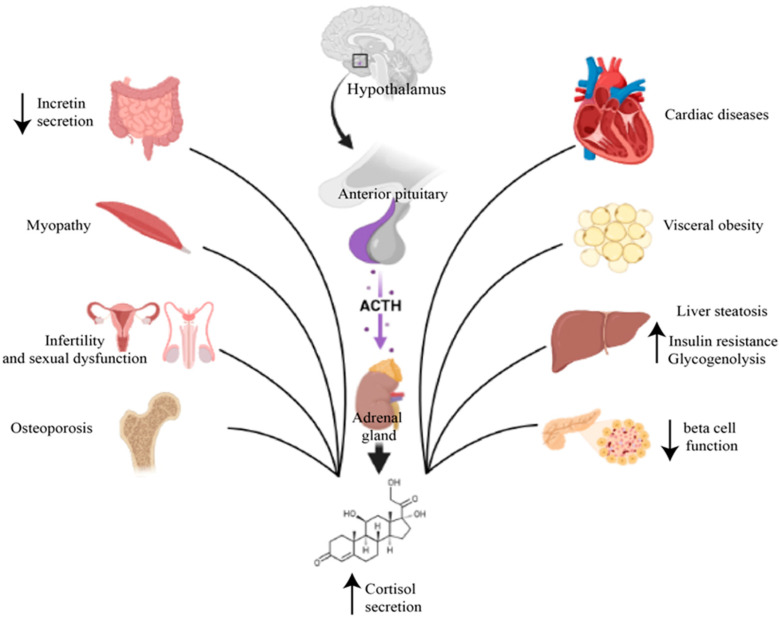
Diabetic implications of Cushing’s syndrome: current therapeutics: Hypothalamus (outlined with a black box), up arrows indicate increase or excess while down arrows indicate decrease. (Figure originally created by the authors using BioRender: Scientific Image and Illustration Software https://www.biorender.com/).

The effects on carbohydrate metabolism are exerted mainly on the level of the liver, skeletal muscles and adipose tissue. At the liver level, excess glucocorticoids stimulate gluconeogenesis by activating numerous genes involved in the hepatic gluconeogenesis process, stimulating lipolysis and proteolysis with increasing substrates for gluconeogenesis, potentiating the action of glucagon and inhibiting glycogenogenesis [43], as shown in Figure 2.

At the muscle level, excess cortisol induces insulin resistance by interfering with different components of the insulin-signaling cascade, as well as by stimulating proteolysis and loss of muscle mass. All this reflects on the reduced capacity of the muscle to synthesize glycogen and capture most of the postprandial glucose from the circulation, for which it is responsible under physiological conditions [44].

Cortisol hypersecretion causes an increase in visceral obesity and a relative reduction in peripheral adipose tissue, with the appearance of central obesity typical of Cushing’s syndrome [45]. Abdominal obesity is closely associated with metabolic syndrome and worsens insulin resistance. Moreover, the hypersecretion of cortisol influences the synthesis and release of hormones from adipose tissue, mainly adipokines, further contributing to the development of insulin resistance [43]. Also, glucocorticoids inhibit the synthesis and secretion of pancreatic insulin, induce pancreatic β cell apoptosis, loss of β cell function and the subsequent development of diabetes [46]. The involvement of the bone system in affecting glucose homeostasis was also found. Long-term exposure to glucocorticoids causes a reduction in circulating osteocalcin that can increase insulin resistance [47].

The first treatment option for Cushing’s syndrome, regardless of etiology, is mainly surgical intervention, either selective pituitary transsphenoidal adenomectomy, unilateral or bilateral adrenalectomy or excision of the ectopic ACTH-secreting tumor. If surgery fails or if surgery is not possible, there are several second-line therapies available. For the cases of postsurgical recurrence or incomplete resection, it is recommended to repeat the surgical procedure or radiotherapy [48].

Drug treatment consists in the administration of steroidogenesis inhibitors, being recommended as a second-line therapy in Cushing’s disease, as the first option in metastatic ACTH ectopic secretion syndrome or if the secreting tumor cannot be located or as an adjunctive treatment for lowering cortisol levels in adrenocortical carcinoma [49].

Due to the fact that cortisol hypersecretion is responsible for changes in carbohydrate metabolism, the normalization of the postsurgical cortisol level generally determines the improvement in glycemic values, but insulin resistance may persist in certain cases, requiring antidiabetic treatment. A study that included 174 patients with Cushing’s syndrome and followed the effects of surgical treatment on hyperglycemia showed that in 47% of cases there was an improvement in blood glucose values, and in 21% of cases, blood glucose returned to normal limits [50].

As in the general population, the treatment of diabetes begins with lifestyle changes that include physical exercise and a diet aimed at reducing the intake of carbohydrates. The drug treatment of first choice is metformin due to its action of reducing insulin resistance. Due to the fact that glucocorticoids mainly exert their effects on metabolism in the postprandial period, GLP-1 receptor agonists and dipeptidyl peptidase 4 inhibitors should be considered as a second treatment option [51,52].

Sodium–glucose cotransporter 2 inhibitors are associated with a higher risk of urinary and genital infections, and the decision to use them must be carefully evaluated in patients with Cushing’s syndrome, who due to the underlying disease already present an increased risk of infection. Insulin may become necessary in patients whose uncontrolled diabetes persists [44].

Patients with Cushing’s syndrome secondary to exogenous corticosteroid administration have a risk of developing diabetes and other complications depending on the dose and duration of corticosteroid administration. For these patients, the treatment must primarily include oral antidiabetics focused on increasing insulin sensitivity and increasing postprandial insulin secretion [43]. Insulin treatment may be necessary if diabetes cannot be controlled with oral medication. Initially, due to the postprandial effects of corticosteroids, only the administration of prandial insulin can be considered [53]. If basal–bolus therapy is adopted, it must be taken into account that patients treated with glucocorticoids require more prandial insulin than basal, in a ratio of 70–30% [54]. Insulin therapy should be considered as a first-line therapy for patients already diagnosed with diabetes who require corticosteroid therapy due to the rapid action of insulin compared to oral antidiabetic agents [43].

## 4. Pheochromocytoma and Diabetes

Pheochromocytoma is a rare catecholamine-secreting tumor that derives from the chromaffin cells of the adrenal medulla. Most pheochromocytomas are benign tumors, but up to 10–15% are malignant [55]. Pheochromocytoma can appear sporadically or within some family syndromes, having an autosomal dominant transmission. The syndromes associated with pheochromocytomas are multiple endocrine neoplasia 2 (MEN 2A and MEN 2B) linked to RET gene mutations, von Hippel–Lindau syndrome due to VHL gene mutations and neurofibromatosis type 1 associated with NF1 gene mutations. MEN2A includes pheochromocytoma, medullary thyroid carcinoma and hyperparathyroidism, and MEN2B represents the association between pheochromocytoma, medullary thyroid carcinoma and neuromas. SDHB gene mutations are frequent in malignant pheochromocytomas [56].

Depending on the type of catecholamine produced, pheochromocytoma can be predominantly noradrenaline-secreting, predominantly adrenaline-secreting or predominantly dopamine-secreting. The effects of catecholamines on the cardiovascular system are the strongest and appear as a result of the stimulation of α receptors with the appearance of peripheral vasoconstriction and of β1 receptors with an increase in contractility and heart rate [57]. The clinical picture of excess catecholamines includes the classic triad: headache, palpitations and sweating. These are accompanied in 80–90% of cases by hypertension, which can be persistent in approximately half of cases, paroxysmal in 45% of cases, and 5–15% of patients are normotensive. Other less common signs and symptoms are anxiety, tremors, abdominal and chest pain, nausea, constipation, weight loss, heat intolerance, pallor, sometimes skin congestion and hyperglycemia [58].

Paroxysmal arterial hypertension is the result of the sudden release of catecholamines and can be induced by triggering factors such as physical exertion, postural changes, anxiety, consumption of foods rich in tyramine (cheeses, wine), pregnancy, surgical interventions and various drugs (tricyclic antidepressants, opioids). The frequency of paroxysms varies from several times a day to once every few months, and their duration varies from a few minutes to over an hour. Episodes of catecholamine discharge can cause heart failure, arrhythmias and intracranial hemorrhages [59]. The diagnosis is established by highlighting an increased level of catecholamines through laboratory analyses and locating the tumor through imaging investigations. Plasma and urinary catecholamines or their metabolites can be dosed directly. The metabolism of catecholamines is achieved under the action of the enzyme catechol-O-methyl transferase, which converts epinephrine to metanephrine and norepinephrine to normetanephrine. Adrenaline and noradrenaline are secreted intermittently, while their metabolites are produced continuously, so the dosing of metanephrines is recommended [57].

The measurement of plasma free metanephrines has a higher sensitivity than the measurement of fractionated free metanephrines from urine over 24 h. When their value is 3–4 times above the upper limit of normal, the diagnosis of pheochromocytoma is very likely. If the results are at the limit, a clonidine suppression test can be performed. False-positive results can occur following the administration of drugs (sympathomimetics, levodopa, MAO inhibitors, tricyclic antidepressants, α and β blockers), coffee consumption or stress [60].

Imaging investigations are usually performed after biochemical confirmation of excess catecholamines. A CT and MRI are also recommended as a first step. If the biochemical measurements confirm the tumor, but the CT or MRI scan does not localize the tumor or if there is a suspicion of a malignant pheochromocytoma with metastasis, a scintigraphy with metaiodobenzylguanidine (MIBG) or a PET-CT with fluoro-deoxy-glucose (PET-FDG) or fluorodihydroxyphenylalanine can be performed (FDOPA PET) [61]. Diabetes mellitus can affect up to 35% of pheochromocytoma patients [62,63].

The excess of catecholamines determines the suppression of insulin secretion at the pancreatic level mainly by stimulating α-adrenergic receptors and insulin resistance in peripheral tissues by stimulating β-adrenergic receptors. At the muscle level, catecholamines inhibit the uptake and use of glucose [64]. At the hepatic level, increases in catecholamine levels increase glucose production by stimulating the processes of glycogenolysis and gluconeogenesis [65]. Surgical resection is the only definitive treatment of pheochromocytoma. Preoperative blood pressure management is extremely important to prevent perioperative cardiovascular complications. The administration of α-receptor blockers is the first treatment option, the most frequently used being phenoxybenzamine. Calcium channel blockers can be used as an alternative. To combat tachycardia and postural hypotension resulting from the use of α blockers, β blockers can be added to the therapeutic regimen but only a few days after α blockers to avoid a hypertensive crisis [57]. Preoperative treatment should be initiated 7–14 days before surgery and should include a sodium-rich diet and increased fluid intake to prevent severe arterial hypotension after tumor removal [61]. Laparoscopic adrenalectomy is the method of choice for most pheochromocytomas. Classical intervention is recommended for large or invasive pheochromocytomas. A partial adrenalectomy with preservation of the adrenal cortex can be performed in the case of patients with hereditary pheochromocytoma and small tumors who have already undergone a complete contralateral adrenalectomy in order to prevent permanent adrenal insufficiency [57].

Tumor-formation resection improves glycemic values. Furthermore, hypoglycemia has been described as a possible postinterventional complication both in diabetic and nondiabetic patients [64]. A study carried out between 1996 and 2015 by Beninato and colleagues, which included 153 patients diagnosed with pheochromocytoma, of which 23% had diabetes or glucose intolerance, showed that after tumor excision, more than 90% of patients presented values of lower blood sugar, and in 79% of cases, the resolution of diabetes was reached. Patients who present other risk factors for the development of diabetes, such as a higher body mass index, are more likely to require antidiabetic medication postoperatively [63]. Another study conducted in 2003 by La Batide-Alanore and collaborators showed that 90% of the diabetic patients included in the study were cured of diabetes. The 10% who were not cured had an incomplete resection of the tumor, with the exception of one patient with hypothyroidism who had a complete resection [62].

**Figure 3:**
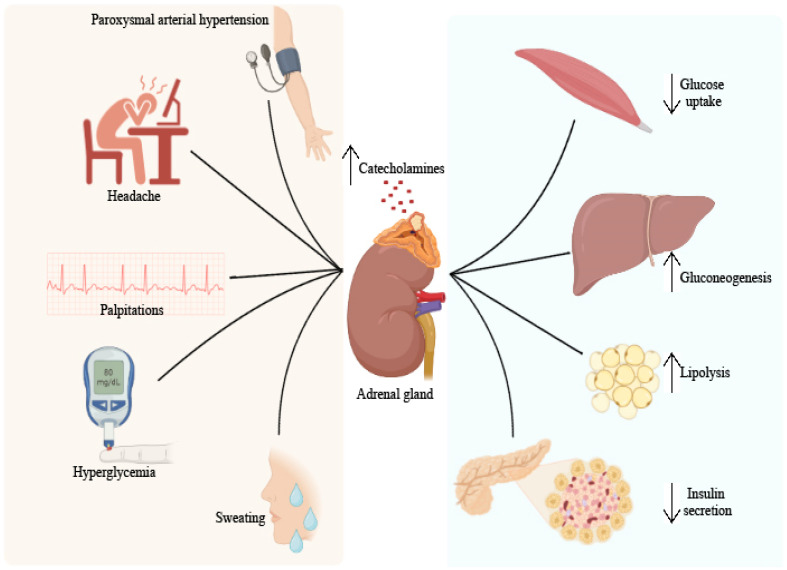
Pheochromocytoma and diabetes: clinical picture of pheochromocytoma and diabetic consequences, up arrows indicate increase while down arrows indicate decrease. (Figure originally created by the authors using BioRender: Scientific Image and Illustration Software https://www.biorender.com/).

The clinical picture of pheochromocytoma and its diabetic consequences are summarized in Figure 3.

## 5. Diabetes Mellitus Secondary to Graves’ Disease

Known as Basedow–Graves’ disease, exophthalmic goiter or von Basedow’s disease, this pathology was first described in the 19th century, through a symptomatological picture that included eye abnormalities, a hyperactive thyroid gland increased in volume and accelerated heart rate [66]. Globally, this thyroid condition is considered the main cause of hyperthyroidism [67]. Although in the first phase the hyperthyroidism of Graves’ disease was considered the result of an increased secretion of thyroid hormones by the pituitary gland, in 1956, through the discovery of antibodies against the TSH receptor, it was established that the pathology belongs to autoimmune diseases [68]. From an epidemiological point of view, Basedow–Graves’ disease reports an annual incidence of 20–50 cases per 100,000 people. Ophthalmopathy associated with Graves’ disease has an incidence of 3 cases per 100,000 men and 16 cases per 100,000 women, with a raised frequency in the Caucasian race [66]. The disease can affect the population in any decade of age, with a maximum incidence between 30 and 50 years, women presenting a risk of approximately 3% of developing this condition during their lifetime. Although they can appear at a long distance from each other, ophthalmopathy and hyperthyroidism usually develop within a year [69].

The triggering of the autoimmune process in Basedow–Graves’ disease starts from a predisposing genetic ground on which environmental factors and endogenous factors act. The autoimmune reaction influences the B lymphocytes of the thyroid gland, which are the basis of the production of antibodies against the TSH receptor, which in most cases, through their stimulating action, will result in thyroid hyperfunction [70].

Several aspects are involved in the clinical manifestations of Graves’ disease, including the severity and period of manifestation of hyperthyroidism but also the age of the patient at the onset of the disease [71]. The signs and symptoms that appear in more than 50% of patients are specific to hyperthyroidism and are characterized by fatigue, heat intolerance, weight loss, palpitations, tachycardia, tremors, anxiety and fatigue [72]. The age of the patient at the onset of the disease is of particular interest because the association of cardiac manifestations, weight loss and loss of appetite with older age has been observed. Atrial fibrillation secondary to hyperthyroidism occurs in more than 10% of patients over 60 years old, and palpable thyroid goiter is associated with an age under 60 years [66].

In young patients under 50 years old, neurological symptoms such as anxiety or tremor predominate in the clinical picture, but cases have been observed in which psychiatric symptoms, such as psychosis, are also associated [73]. Orbitopathy from Basedow–Graves’ disease can be present in different stages, it can be limiting, it affects vision, and it is characterized by lacrimation, inflammation, irritation, retroorbital pain and exophthalmos [66]. Rundle and associates described for the first time the clinical model according to which Graves’ orbitopathy develops. Initially, an active phase of the disease appears, which extends over a period of approximately 3 years and is characterized by inflammation and congestion. In the active phase, ocular clinical manifestations may appear, including proptosis, diplopia, hyperlacrimation, pain, ocular discomfort, dry eye and periorbital edema. An inactive phase follows, in which the manifestations of orbitopathy persist but are stable [74].

The dermatopathy associated with Basedow–Graves’ disease most frequently associates severe cases of ophthalmopathy, and although it can also appear in other areas such as elbows and fingers, it is most frequently located pretibial [68]. In patients with dermatopathy, acropachia may appear, which is similar to clubbing [75]. Basedow’s disease diagnosis is based on the presence of clinical signs and symptoms and laboratory investigations. The Merseburg triad consisting of diffuse gout, thyrotoxicosis and ophthalmopathy raises a clear suspicion of the disease. From the point of view of laboratory analyses, this disease is characterized by undetectable levels of TSH and increased levels of triiodothyronine T3 and thyroxine T4. As additional tests, TSH receptor antibodies (TRabs) can be dosed [72].

The radioactive iodine absorption test (RAIU) and the thyroid scintigram that reveals an enlarged thyroid with a homogeneous capture of I-123 are also important in establishing the diagnosis [76,77]. Hyperthyroidism from Graves’ disease is treated either by using synthetic antithyroid drugs or by using radioiodine therapy or by resorting to a thyroidectomy. Synthetic antithyroid drugs are the first-line treatment, especially in the case of young, pregnant or short-term patients before a thyroidectomy or radioiodine therapy. This category includes carbimazole, methimazole and propylthiouracil [78]. They all block the synthesis of thyroid hormones by inhibiting the organization of iodine. Propylthiouracil in high doses also blocks the peripheral conversion of T4 into T3. The duration of the treatment is 12-18 months, after which the treatment can be interrupted if the concentrations of TSH and TRAb reach normal values [79]. Beta blockers can be used in the initial treatment of Graves’ disease, before thyroid function is normalized, to reduce adrenergic symptoms. Propranolol or other long-acting beta blockers are preferable [80].

Treatment with radioactive iodine causes the progressive destruction of thyroid tissue and is recommended in the case of relapses after treatment with synthetic antithyroid drugs or in the event of severe side effects of synthetic antithyroid drugs. Pregnancy, breastfeeding, severe ophthalmopathy and the presence of nodules with a risk of malignancy are contraindications [78]. A total or subtotal thyroidectomy is indicated for patients with large, compressive goiters, in case of a suspicion of malignancy or if the evolution is unfavorable under antithyroid treatment [81].

The treatment of Graves’ ophthalmopathy consists primarily in quitting smoking and restoring the euthyroid state. For the therapy of mild forms, it is recommended to take supplements based on selenium, as well as local treatment measures (artificial tears, ophthalmic ointments), and for moderate to severe forms, IV corticosteroid therapy in high doses as a pulse therapy is recommended [82]. Orbital radiotherapy is preferred in patients with severe ocular mobility disorders, while surgical decompression is recommended 6 months after the inactivation of the disease in moderate to severe forms [81].

There are very few data in the literature regarding the frequency of diabetes secondary to Basedow–Graves’ disease. A study conducted by Song and associates with the aim of evaluating the risk of diabetes mellitus in patients with Basedow–Graves’ disease included 4593 patients with Graves’ disease. Diabetes was diagnosed in 16.3% of patients over a 7-year follow-up period. Moreover, the study showed that patients treated with radioactive iodine had a higher risk of developing diabetes than those treated with synthetic antithyroid drugs, and the risk increases with the increase in the duration of treatment [83]. The excess of thyroid hormones contributes to the occurrence of diabetes both by affecting insulin secretion and by increasing insulin resistance at the peripheral level [2]. Hyperthyroidism is associated with an increased level of postprandial plasma insulin and proinsulin but also with an increased apoptosis of pancreatic β cells. Moreover, the half-life of insulin is reduced probably due to the increased rate of degradation and the high release of biologically inactive insulin precursors [84].

In untreated Graves’ disease, proinsulin levels in response to food intake increase [85]. At the level of the pancreatic α cells, the effect of thyroid hormones translates into the stimulation of glucagon secretion. Hyperthyroidism causes the acceleration of intestinal peristalsis and an increased absorption of glucose from the intestine [86].

Thyroid hormones increase hepatic glucose production by increasing the hepatic expression of the glucose transporter GLUT 2 and stimulate endogenous glucose production by increasing gluconeogenesis and glycogenolysis processes, leading to a decrease in liver sensitivity to insulin [87]. Also, hyperthyroidism stimulates lipolysis, resulting in an increase in the levels of free fatty acids and consequently the stimulation of hepatic gluconeogenesis, and at the muscle level, it causes an increase in GLUT 4 expression and the use of glucose [88].

The treatment of hyperthyroidism with synthetic antithyroid drugs does not significantly affect glycemic control. In the study conducted by Maxon et al. (1975), 40% of patients presented disturbances in carbohydrate metabolism 9 months after obtaining euthyroidism, and in another study conducted in India in 2019, a third of patients had altered glucose tolerance 3 months after obtaining euthyroidism [89,90] There are no specific recommendations for the treatment of diabetes secondary to Graves’ disease. However, certain oral antidiabetics can influence aspects of Graves’ disease.

**Figure 4:**
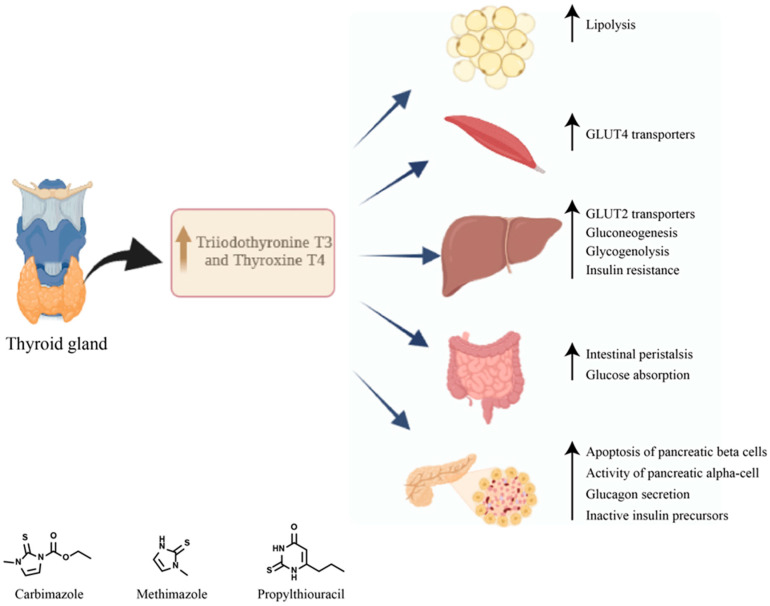
Diabetes mellitus secondary to Graves’ disease: diabetic complications and therapeutics of Graves’ disease. Up arrows indicate increase or excess while down arrows indicate decrease (figure originally created by the authors using BioRender: Scientific Image and Illustration Software https://www.biorender.com/).

Thiazolidinediones should be used with caution in Graves’ disease and even avoided in patients with active Graves’ orbitopathy. Thiazolidinediones, as agonists of the PPARγ nuclear receptor expressed in orbital adipose and connective tissues, stimulate the differentiation of adipocytes at the orbital level and aggravate ophthalmopathy [91]. Also, the administration of metformin to patients with insulin resistance was associated with a reduction in the volume of the thyroid gland but also in the size of the thyroid nodules [92]. Figure 4 summarizes the diabetic complications and therapeutics of Graves’ disease.

## 6. Diabetes Mellitus Secondary to Primary Aldosteronism

Primary aldosteronism is an endocrinological disease, considered the most common cause of resistant hypertension, being associated over the years with an increased risk of cardiovascular complications [93]. Primary aldosteronism is characterized by a hypersecretion of aldosterone in the context of a low pasmatic renin. With a prevalence of 20% among patients with resistant HTN and 10% among patients with severe HTN, once this pathology was considered rare, being discovered and treated in a relatively low percentage [94]. In the pathophysiology of AP, the renin–angiotensin–aldosterone system is involved, an essential hormonal cascade in the homeostasis of hydroelectrolytic balance and blood pressure regulation [95].

Glycoprotein hormone (secreted at the level of juxtaglomerular cells from the level of the renal afferent arteriole) renin cleaves the circulating angiotensinogen into angiotensin I, which ends up being converted with the help of the conversion enzyme to angiotensin II. Through the AT1 type 1 receptor, angiotensin II induces renal sodium resorption, aldosterone secretion and vascular contraction. Aldosterone acts on kidney cells by increasing sodium reabsorption, induces potassium secretion and can cause injuries, inflammation or fibrosis at the cardiac, renal or blood vessel levels [96]. Angiotensin II and potassium secretion normally stimulate aldosterone production, but in this pathology, aldosterone production is independent of the renin–angiotensin–aldosterone system, is inappropriately high and cannot be suppressed by sodium loading. The main causes of primary hyperaldosteronism are idiopathic aldosteronism with bilateral adrenal hyperplasia and unilateral aldosterone-secreting adenoma [97].

From a biochemical point of view, PA is characterized by the suppressed activity of plasma renin, hypersecretion of aldosterone, and although hypokalemia can be observed in most patients, studies show that only a percentage of 9–37% of patients present this change [98]. Hypertension secondary to primary aldosteronism occurs based on the activation of the sympathetic nervous system and hypervolemia. It has been observed, however, that edema rarely occurs in this pathology, hypertension from aldosteronism being similar to primary arterial hypertension [99].

The risk of cardiovascular events is much higher in patients with primary aldosteronism compared to those with primary essential hypertension. Hyperaldosteronism can increase the risk of myocardial infarction up to 6 times, the risk of stroke up to 4 times or the risk of atrial fibrillation up to 12 times. In addition, patients are exposed to an increased risk of left ventricular hypertrophy, tissue fibrosis or increased stiffness of the large arteries. The identification and initiation of treatment are essential to prevent cardiovascular and cerebral consequences [100]. Data published in recent years demonstrate the association of primary aldosteronism with an increased risk of stroke, coronary heart disease, heart failure, arterial fibrillation and CVD but also diabetes and metabolic syndrome compared to people diagnosed with essential hypertension [3,101]. Insulin sensitivity in skeletal muscles and adipocytes is affected by mineralocorticoid receptor activation induced by aldosterone [102].

Studies show that aldosterone is secreted in excess in patients suffering from obesity. Since obesity is a main risk factor for type 2 diabetes, hyperaldosteronism in obese patients can contribute to a change in glucose tolerance both by affecting insulin secretion and by changing insulin sensitivity [103]. Hypokalemia has been shown not to be specific to the disease; however, in cases of hypokalemia, it can be accompanied by metabolic disorders such as hypomagnesemia, mild hyponatremia or alkalosis [104]. Screening for hyperaldosteronism is extremely important, due to the increasing prevalence of this pathology and its known impact on cardiovascular morbidity. Biochemical testing by measuring the ratio between plasma aldosterone concentration (PAC) and plasma renin activity (PRA), also known as ARR, is a sensitive and useful marker in screening because it detects an increase in plasma aldosterone in combination with a low renin level, thus being superior to the isolated dosage of potassium, DRC, or serum aldosterone [105].

After interpreting the ARR screening test as positive, it is necessary to establish the diagnosis of primary hyperaldosteronism. The purpose of the test is to evaluate aldosterone secretion, whether it is suppressible or not, because in true primary hyperaldosteronism it cannot be suppressed [106]. For the diagnosis of primary hyperaldosteronism, four confirmatory tests were approved: oral sodium loading, fludrocortisone suppression test (FST), captopril test and saline infusion test (SIT). Each test offers specific advantages or disadvantages, so there is no optimal test which can be considered the “gold standard” in the diagnosis of this pathology [107].

Hypertension secondary to primary aldosteronism occurs based on the activation of the sympathetic nervous system and hypervolemia. It has been observed, however, that edema rarely occurs in this pathology, hypertension from aldosteronism being similar to primary arterial hypertension [99]. The risk of cardiovascular events is much higher in patients with primary aldosteronism compared to those with primary essential hypertension. Hyperaldosteronism can increase the risk of myocardial infarction up to 6 times, the risk of stroke up to 4 times or the risk of atrial fibrillation up to 12 times. In addition, patients are exposed to an increased risk of left ventricular hypertrophy, tissue fibrosis or increased stiffness of the large arteries. The identification and initiation of treatment is essential to prevent cardiovascular and cerebral consequences [100]. Data published in recent years demonstrate the association of primary aldosteronism with an increased risk of stroke, coronary heart disease, heart failure, arterial fibrillation and CVD but also diabetes and metabolic syndrome compared to people diagnosed with essential hypertension [101]. Although the mechanism remains unclear, ever since the initial description of hyperaldosteronism by Dr. Conn in 1950, excess aldosterone has been associated with the onset of diabetes. Hypokalemia secondary to hyperaldosteronism was initially described as a reason for the change in glucose tolerance, by affecting insulin secretion. However, the administration of potassium to correct hypokalemia has managed to normalize insulin secretion and glucose tolerance only partially [3,108].

Insulin sensitivity in skeletal muscles and adipocytes is affected by mineralocorticoid receptor activation induced by aldosterone [102]. Studies show that aldosterone is secreted in excess in patients suffering from obesity. Since obesity is a main risk factor for type 2 diabetes, hyperaldosteronism in obese patients can contribute to a change in glucose tolerance both by affecting insulin secretion and by changing insulin sensitivity [103]. It is suggested that aldosterone affects insulin sensitivity via the mineralocorticoid receptor (MR) in both humans and rodents. Moreover, a prediction of the development of insulin resistance in 10 years was observed, in people with elevated plasma aldosterone, suggesting a correlation between hyperaldosteronism and a potential onset of type 2 diabetes [109]. From a clinical point of view, patients evaluated for the suspicion of primary hyperaldosteronism have, of course, long-lasting hypertension, in most cases refractory to more and more drugs. Although this pathology is often symptomatic, when symptoms are present, they are generally attributed to hypokalemia and hyperaldosteronism. Among the main symptoms are fatigue, weakness, muscle–skeletal spasms, headache, polyuria and polydipsia [106]. Hypokalemia has been shown not to be specific to the disease; however, in cases of hypokalemia, it can be accompanied by metabolic disorders such as hypomagnesemia, mild hyponatremia or alkalosis [104].

Screening for hyperaldosteronism is extremely important, due to the increasing prevalence of this pathology and its known impact on cardiovascular morbidity. Biochemical testing by measuring the ratio between plasma aldosterone concentration (PAC) and plasma renin activity (PRA), also known as ARR, is a sensitive and useful marker in screening because it detects an increase in plasma aldosterone in combination with a low renin level, thus being superior to the isolated dosage of potassium, DRC, or serum aldosterone [105]. After interpreting the ARR screening test as positive, it is necessary to establish the diagnosis of primary hyperaldosteronism. The purpose of the test is to evaluate aldosterone secretion, whether it is suppressible or not, because in true primary hyperaldosteronism it cannot be suppressed [106].

For the diagnosis of primary hyperaldosteronism, four confirmatory tests were approved: oral sodium loading, fludrocortisone suppression test FST, captopril test and saline infusion test (SIT). Each test offers specific advantages or disadvantages, so there is no optimal test, which can be considered the “gold standard” in the diagnosis of this pathology [107]. The last step of the diagnostic procedure is represented by the localization of primary aldosteronism in order to distinguish a unilateral effect from a bilateral one. This step is essential in establishing a potential treatment: unilateral laparoscopic adrenalectomy in the case of unilocalized adenomas or mineralocorticoid receptor antagonists in the case of a bilateral disease [110]. The gold standard for locating the disease is currently represented by adrenal venous sampling (AVS), superior to CT but with limitations in medical practice due to an invasiveness that leads to risks such as adrenal venous rupture, patient discomfort and the need of experienced interventional radiologists [111].

A noninvasive but useful tool in the subtyping of primary hyperaldosteronism is represented by high-resolution computed tomography. The results obtained after this evaluation can be either adrenal glands with a normal appearance, minimal unilateral thickening of the adrenal glands, unilateral macroadenoma or bilateral adenomas. However, the functionality of the detected unilateral masses cannot be evaluated by computed tomography [112].

The treatment of primary aldosteronism is aimed at improving or normalizing arterial hypertension and correcting hypokalemia. However, due to the increased risk of cardiovascular diseases or type 2 diabetes, it is necessary to focus the treatment on decreasing aldosterone production or blocking the mineralocorticoid receptor [113].

In the guidelines of the Endocrine Society for the management of primary aldosteronism, unilateral laparoscopic adrenalectomy is recommended for patients with a unilateral disease, or treatment with MRAs (mineralocorticoid receptor antagonists) is recommended for patients who do not want or cannot undergo surgery but also in the case of bilateral damage. It is recommended to initiate first-line therapy with spironolactone or, as an alternative, eplerenone [93]. Spironolactone in the maximum dose that allows control of hypokalemia and blood pressure is the diuretic of choice in the medical treatment of primary aldosteronism. Eplerenone has proved to have fewer adverse effects and a greater selectivity for mineralocorticoid receptors than spironolactone, but it is still less potent and is not approved for the treatment of this pathology in the USA or Europe [113].

There are a multitude of studies that show an improvement in arterial hypertension after adrenalectomy [114,115]. Wu and associates describe in a longitudinal study a reduction in mortality from various causes in the case of resorting to total adrenalectomy in favor of treatment with mineralocorticoid receptor antagonists in monotherapy in patients with unilateral primary aldosteronism [116]. The Taiwan Primary Aldosteronism Investigation also highlights, in a study conducted on 20 patients with primary aldosteronism, an improvement in myocardial fibrosis but also an improvement in arterial stiffness and intima–media thickness after surgical intervention [117]. A study conducted by Komada and colleagues concluded a reduction in aldosterone plasma levels and an improvement in hypokalemia after surgical treatment of the tumor. They also determined an improvement in total insulin secretion but also in the glycemic index, measured during the glucose tolerance test, after the surgical intervention [118]. Another study that aimed to observe the change in glucose tolerance and insulin sensitivity after the treatment of primary aldosteronism observed the rapid and long-term restoration of insulin sensitivity both in patients who underwent surgery and in those who were subjected to drug therapy [119].

## 7. Diabetes Mellitus and Somatostatinoma

Somatostatin-secreting tumors known as somatostatinomas are rare neuroendocrine tumors, with an incidence of approximately 1 in 40 million people and represent approximately 4% of gastrointestinal neuroendocrine neoplasms. They are clinically characterized by weight loss, diarrhea, steatorrhea, gallstones, hypochlorhydria and diabetes [120]. The main peptide released by somatostatinomas is represented by somatostatin, a hormone isolated in 1973 by the research group of Nobel Prize-winning endocrinologist Roger Guillemin [121]. Initially isolated from the hypothalamus, and called the GH release-inhibiting hormone, somatostatin was later determined to be produced and secreted also at the pancreatic level, in the islets of Langerhans.

The two main forms of somatostatin in the body, somatostatin 28 (SRIF28) and somatostatin 14 (SRIF14), derive from preprosomatostatin, which is cleaved into prosomatostatin. Pancreatic delta cells secrete somatostatin 14, while the dominant secretory form at the level of the gastrointestinal tract is somatostatin 28 [122].

Somatostatin is a hormone secreted by the delta cells of the pancreas, stomach and intestine and has a paracrine role of inhibiting the secretion of glucagon and insulin from the pancreatic islet cells [123]. Following the inhibition of insulin secretion by the increased serum values of somatostatin, patients with somatostatinoma can present hyperglycemic changes through glucose intolerance from modified fasting glucose to the onset of diabetes [124]. By inhibiting the secretion of glucagon, growth hormone and other hormones that are capable of increasing blood sugar, it is assumed that somatostatinoma can also have a hypoglycemic action, although the mechanism by which hypoglycemia is produced is not yet sufficiently studied. In 1980, Wright and associates presented the first clinical case of a woman with somatostatinoma accompanied by hypoglycemia at presentation, who during treatment presented with both hypoglycemia and hyperglycemia [125].

Somatostatin inhibits the release of medium pancreatic enzymes and cholecystokinin. As a result of these effects, the hypersecretion of somatostatin will lead to the development of diabetes, steatorrhea, gallstones (following the decrease in the contractility of the gallbladder) and malabsorption [126]. Numerous secretory functions, both endocrine and exocrine, are under the control of somatostatin. Almost all intestinal hormones are inhibited by somatostatin, including gastrin, secretin, GIP (gastric inhibitory polypeptide), insulin and glucagon, but the direct effects of the hormone on certain target organs are also known. It has effects on intestinal motility, is involved in the absorption of nutrients in the small intestine and inhibits the secretion of gastric acid and pancreatic enzymes [127]. Somatostatinomas represent less than 5% of functional pancreatic neuroendocrine tumors and are formed by somatostatin-secreting delta cells, which result in a hypersecretion of this hormone. In addition to the pancreatic location, they can also appear at the duodenal level, especially periampullary. True somatostatinomas are rare and most of the time are discovered accidentally following investigations for other pathologies [128]. These tumors can be sporadic, but most often, they can appear in association with other syndromes, most frequently involved in multiple endocrine neoplasia type I, Von Hippel–Lindau syndrome or neurofibromatosis type 1 [129].

From a genetic point of view, chromosomal aberrations such as the loss of heterozygosity on chromosome 11q, 6q and the modification of chromosome 3p alleles have implications in the pathogenesis of this disease. Regarding age and gender, somatostaninoma most frequently appears late in adults after their 40s, without a gender predilection [130]. Nonfunctional somatostatinoma is frequently found at the level of the duodenum and is discovered either accidentally or following the presentation of the patient for symptoms related to the mechanical obstruction of the biliary tree [131]. This can determine, in the evolution of the disease, a clinical picture characterized by jaundice or abdominal pain [132].

Secretory or functional tumors are most often found in the pancreas and describe a specific clinical picture given by the effects of somatostatin on the body. In symptomatic cases, cholelithiasis occurs in 70% of cases and diabetes in 60% [130]. Diabetes mellitus was observed in 75% of patients with somatostatinoma of a pancreatic localization, but as regards the duodenal localization, only 11% of them presented this pathology. Diabetes secondary to somatostatinoma is well controlled with oral antidiabetics and diet or, in some cases, with low doses of insulin. The absence of ketoacidosis is explained by the inhibitory role of somatostatin for both insulin and glucagon. A cause for the increased incidence of diabetes in patients with pancreatic tumors compared to duodenal tumors could be the replacement of the functional tissue at this level with tumor cells [133].

There are rare cases in which an inhibitory syndrome appears, caused by the suppression of insulin, exocrine pancreatic enzymes and cholecystokinin, characterized by the triad of diabetes mellitus, steatorrhea and cholelithiasis [134]. Regarding the early diagnosis of somatostatinoma, this is often made difficult either by the lack of symptoms in the case of nonfunctional tumors or by nonspecific symptoms that point us toward other conditions in the case of secretory tumors [130].

The fasting serum level of somatostatin is usually increased in this pathology, but this dosage is not specific because somatostatin also increases in pheochromocytoma, lung cancer, medullary thyroid carcinoma or paraganglioma [135]. As an alternative, in some centers, the 24 h urine level of 5-hydroxy indole acetic acid (5-HHIA), which is a breakdown product of serotonin, was measured, but certain factors can interfere with its measurement, such as certain medicines and food products [136]. Paraclinical investigations that can be used to establish a diagnosis are computed tomography with a contrast of the abdomen (considered the choice due to noninvasiveness and wide availability) or MRI, which has a greater sensitivity than CT for the detection of small secondary liver lesions [126].

To view and take biopsies in order to confirm the diagnosis, endoscopic ultrasound (EUS) is used/indicated in tumors at the level of the duodenal wall, or at the level of the head of the pancreas, endoscopic retrograde cholangiopancreatography (ERCP) or esophagogastroduodenoscopy (EGD) [130]. The only curative treatment is represented by surgical intervention, with the excision of the tumor but also of all metastasized lymph nodes [137]. Preferred procedures include distal pancreatectomy with splenectomy, proximal pancreatoduodenectomy with preservation of the folds or the Whipple procedure [138]. Tumor staging is essential in order to initiate surgical treatment because grade 3 neuroendocrine tumors are most often metastasized and become inoperable [139]. For patients with inoperable tumors or metastases, somatostatin analogs present an important treatment option in order to improve symptoms. They represent the first line in palliative treatment for controlling somatostatin secretion and tumor growth [140].

Controlled studies showed that the administration of 120 mg of lanreotide every 4 weeks was associated with a prolonged survival of patients compared to the placebo group [141]. Somatostatin analogs used in the treatment of neuroendocrine tumors are frequently associated with adverse effects such as hyperglycemia. Studies conducted on 29 patients with metastatic pancreatic neuroendocrine tumors who were treated with pasireotide LAR 60 mg/month report a significant increase in fasting blood glucose from 106 mg/dL at the beginning of the study to 150 mg/dL at its end [142]. Although in the specialized literature there are no guidelines dedicated to the therapy of metabolic diseases secondary to endocrinopathies, associated diabetes can usually be controlled with oral agents and rarely requires the use of insulin [143].

## 8. Diabetes Mellitus Secondary to Glucagonoma

**Figure 5:**
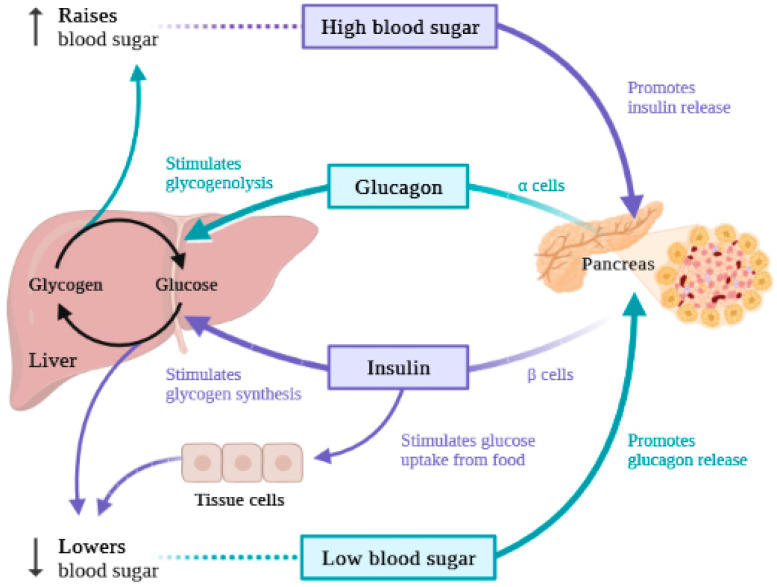
Regulation of blood glucose level by insulin/glucagon balance (figure originally created by the authors using BioRender: Scientific Image and Illustration Software https://www.biorender.com/).

Glucagon is a hormone secreted by the alpha cells of the pancreas, in response to a decrease in the concentration of glucose in the body in certain situations such as prolonged fasting or intense physical exertion. It consists of 29 amino acids and is derived from a peptide called proglucagon. By stimulating gluconeogenesis and glycogenolysis, glucagon is known for increasing blood glucose levels, but it also plays a role in amino acid metabolism. Insulin resistance, which results in diabetes, is a result of a long-term exposure to elevated blood glucagon levels [144], as depicted in Figure 5. Besides diabetes, a whole spectrum of metabolic complications, such as cardiovascular complications or obesity, are associated with glucagon secretion and insulin resistance because they are closely related to the control of body weight and energy consumption [145]. Along with insulin, glucagon also promotes ketogenesis, contributing to maintaining a healthy and necessary fuel balance for a harmonious functioning of the body. In other words, glucagon is a hormone that mobilizes glucose, while insulin functions as an anabolic hormone [146].

Most people diagnosed with type 2 diabetes develop hyperglucagonemia during fasting, but this cannot be considered a general response because there are patients in whom no change is observed. It has been hypothesized that the initial lack of glucagon suppression or hypersecretion during a high-carbohydrate meal indicates that the cellular response to hyperglycemia is diminished, although the exact mechanism underlying this impact is still unclear [147,148].

Among the main causes of hyperglucagonemia are the postoperative state of gastric bypass, nonalcoholic fatty liver disease and glucagonoma [149]. Pancreatic neuroendocrine tumors represent a group of tumors with varied tumor biology, prognosis and clinical presentation. Glucagonoma is among pancreatic neuroendocrine tumors (pNETs) that include heterogeneous functional neoplasias [150].

Glucagonoma is a rare, hormonally active neuroendocrine tumor that results in glucagon hypersecretion. Clinical manifestations are defined as glucagonoma syndrome (GS). Excess glucagon is provided by the appearance of tumors in alpha pancreatic cells, tumors that usually have a slow growth rate and are called functional pancreatic tumors [151].

In 1942, Becker and colleagues described the first case of glucagonoma in a 45-year-old woman, whose symptoms were weight loss, glossitis, extensive dermatitis and impaired glucose tolerance. After the autopsy, a neoplasm with islet cells in the pancreas was also revealed [152].

McGavran and his colleagues identified in 1966 a metastatic tumor of the pancreatic islets, which was associated with the clinical picture present in diabetes and dermatitis but also with high levels of glucagon in the blood [153,154]. Another study carried out by Yao and associates that aimed to evaluate the epidemiology and survival prognosis in patients with islet cell carcinoma describes a 2% incidence of patients with glucagonoma out of 1310 cases of pancreatic tumors of neuroendocrine origin [155].

Glucagonomas are often large tumors, with dimensions at diagnosis of approximately 3 cm which, due to the high concentration of alpha cells present in the tail and body of the pancreas, develop most frequently at this level. At the time of diagnosis, more than half of the tumors have metastases. The frequency in men and women is approximately equal, and as for the age of onset, most patients present symptoms in the sixth decade of life.

The estimated global incidence of glucagonoma is approximately 1 in 20 million people, so we can talk about a rare syndrome [156]. Kindmark and associates reported in a study conducted on patients with pancreatic tumors with glucagon hypersecretion, diagnosed over a period of 20 years, the occurrence of glucagonoma in approximately 7% of the subjects included in the study (23 out of 340). Of these, 22% had a diagnosis of diabetes before the appearance of the glucagonoma, and 35% developed diabetes during the study period [152,157]. In a study carried out on 623 cases (268 men and 339 women) with the aim of providing new data regarding the clinical picture, diagnosis and treatment of glucagonoma, the following clinical findings were observed: necrolytic migratory erythema was present in 82.4% of subjects, diabetes in 68.5%, anemia in 49.6%, weight loss was observed in 60.2% and mucosal damage in 41.2% of patients [158].

Depression can occur in approximately 50% of patients and is considered a consequence of chronic dermatopathy. Besides this, other neuropsychiatric manifestations include psychosis, dementia, ataxia, paranoid delusions, agitation and proximal muscle weakness [152]. Although the exact cause of erythema migrans is not exactly known, it is suspected to be a result of a combination of inadequate nutrition and a low level of zinc and amino acids in the body. Diabetes occurs secondary to the direct effects of glucagon. Gastrointestinal disorders, such as diarrhea, may occur as a result of glucagon hypersecretion associated with gastrin, VIP, serotonin or calcitonin secretions [152].

The most common skin manifestation in this pathology is represented by necrolytic migratory erythema. It can be present in up to 70% of cases [159]. The erythema is widespread, and the main affected areas include the perigenital, perioral or extremities. It is initially characterized by erythematous, painful plaques or papules, which increase in size gradually until they end up forming bullous lesions [152]. The main processes involved in necrolytic migratory erythema are represented by superficial epidermal necrosis and the formation of crusts and vesicles but also healing through hyperpigmentation. Following a skin biopsy, vacuolated or dyskeratotic keratinocytes, necrosis of the upper epidermis, pallor of keratinocytes and psoriasiform hyperplasia can be highlighted [160]. The hypersecretion of glucagon with its catabolic effect, in association with the malnutrition resulting from diarrhea, can result in the onset or worsening of already established diabetes. Hyperglycemia in glucagonoma is considered to be closely related to the size of the tumor [161]. The diagnosis of this pathology includes both the clinical findings, including the appearance of necrolytic migratory erythema, and the documentation of elevated serum glucagon levels but also the evidence of a glucagon-secreting tumor from the islet cells of the pancreas [162].

Fasting glucagon dosing is necessary in the pathology of pancreatic tumors. Increased levels of glucagon in the blood can be considered diagnostic criteria for glucagonoma; however, hypergluconemia can also occur in other pathologies such as acute or chronic pancreatitis, liver cirrhosis, renal failure, diabetes, Cushing’s syndrome, stress, trauma and chronic liver failure [151]. The gold standard in diagnosis is the visualization of the tumor by angiography, a procedure with an important role in the visualization of liver metastases. Due to the availability and costs of medical practice, the first-line tools are ultrasound and computed tomography. Other paraclinical methods of investigation include magnetic resonance imaging (MRI) or PET-CT [163,164]. Changes in blood tests, such as HbA1c, serum insulin, C-peptide, serum glucose or glycoalbuminemia can highlight an impaired glucose tolerance. It is known that the size of the tumor and the occurrence of metastases are directly related to the increase in serum glucose. The level of zinc in the blood is also low, and the assessment of liver function, especially the dosage of alkaline phosphatase, is also important for a more complete picture [151].

Nonspecific biochemical markers such as chromatogranin A, a marker for well-differentiated neuroendocrine tumors, pancreatic polypeptide, which is a marker for nonfunctional pancreatic tumors, or neuron-specific enolase, a marker for poorly differentiated tumors, can be dosed to monitor tumor progression but also for diagnosis [165].

Regarding the treatment of glucagonoma, if, at the time of diagnosis, the tumor is localized, surgical resection is the only curative option. The type of resection must be determined according to the location and size of the tumor, after a careful and complete evaluation [166].

Complete oncological resection in the case of glucagonoma includes total pancreatectomy in the case of glucagonoma spread over the entire surface of the pancreas, pancreatoduodenectomy indicated for tumors limited to the head of the pancreas or distal pancreatectomy with or without splenectomy for tumors in the tail or body of the pancreas [167]. Prior to surgical resection, patients must be administered heparin in order to prevent venous thrombosis and initiate parenteral nutrition and glycemic control [168]. However, since at the time of diagnosis between 50% and 90% of glucagonomas have metastases, surgical removal is not always possible [169]. In patients with surgical contraindications, chemotherapy with streptozicin, fluorouracil, doxorubicin, chlorozotocin, dacarbazine, irinotecan, etoposide or taxanes can be used because they are substances that, when used selectively, lead to the destruction of islet cells [170].

Somatostatin analogs, especially octeotride but also lantreotide, have a role in lowering the serum concentration of glucagon, help to limit the tumor and have demonstrated a real success in resolving necrolytic migratory erythema [171]. Somatostatin analogs target the somatostatin receptors that are overexpressed in patients with glucagonoma and thus manage to achieve a reduction in the release of glucagon in the blood and a shrinking of the tumor, which leads to an improvement in symptoms such as hyperglycemia or necrolytic migratory erythema [172].

Diabetes is seen in two-thirds of glucagonoma patients. Generally, there is only mild diabetes or abnormal glucose tolerance. Noninsulin-dependent diabetes mellitus is more common, without obvious complication or ketoacidosis. Although the serum glucagon level does not always coincide with the presence of diabetes, it is possible to explain how excessive amounts of glucagon may cause a malfunction of glucose metabolism. In most situations, diet, oral hypoglycemic medications, or, in rare circumstances, insulin can be used to treat diabetes in glucagonoma syndrome [173].

We summarize all the endocrine diseases mentioned in this article and the recommended treatment for each pathology in Table 1.

## 9. Conclusions

Diabetes is a chronic, complex disease with a vast clinical picture and complications involving several organs and systems. This pathology requires permanent follow-up and appropriate treatment, aiming at both glycemic control and the prevention of the risk factors involved in its pathogenesis. Diabetes mellitus secondary to endocrine diseases is generally characterized by mild signs and symptoms, with hyperglycemia that is most often reversible after the therapy of the underlying disease. The importance of screening for diabetes in endocrine diseases lies in the fact that diabetes increases the risk for cardiovascular mortality, and its early detection can improve the quality of life of patients. The treatment of diabetes secondary to endocrine diseases is generally the treatment of the underlying endocrine disease, either through surgical interventions or through drug treatment, but also includes the therapeutic modalities initiated in the treatment of type 1 or 2 diabetes. The glycemic control of secondary diabetes, either through a hygienic–dietetic regime, oral antidiabetics or insulin therapy, is largely managed according to the same rules and principles as the therapy for primary forms of diabetes.

## Figures and Tables

**Table 1:** Endocrine diseases that can lead to secondary diabetes mellitus with the recommended therapy.

Disease	Treatment	References
**Acromegaly**	Transsphenoidal surgical resection of the GH-secreting adenoma	[21]
	2. Somatostatin analogs (octreotide, lanreotide, pasireotide)	[8,22,23]
	3. Radiation therapy	[20]
	4. Growth hormone (GH) receptor antagonist (pegvisomant)	[8]
	5. Dopaminergic agonists (carbegoline, bromcriptine)	[29]
**Cushing’s Syndrome**	Surgical intervention such as selective pituitary transsphenoidal adenomectom, unilateral or bilateral adrenalectomy or excision of the ectopic ACTH-secreting tumor	[48]
	2. Radiotherapy (postsurgical recurrence or incomplete resection)	[48]
	3. Steroidogenesis inhibitors	[49]
**Pheochromocytoma**	Curative Laparoscopic adrenalectomy Classical intervention for invasive tumors Partial adrenalectomy with preservation of the adrenal cortex	[61]
	2. Preoperative: α-receptor blockers (phenoxybenzamine), calcium channel blockers, beta blockers	[65]
**Graves’ disease**	Synthetic antithyroid drugs (carbimazole, methimazole, propylthiouracil)	[78,79]
	2. Thiroidectomy	[81]
	3. Radioiodine therapy	[78]
	4. Beta blockers: propanolol	[80]
	5. Selenium supplements	[82]
**Primary aldosteronism**	Unilateral laparoscopic adrenalectomy (unilateral disease)	[112]
	2. Mineralcorticoid receptor antagonist: spironolactone, eplerenone (bilateral disease)	[113]
**Somatostatinoma**	Curative: distal pancreatectomy with splenectomy or proximal pancreato-duodenectomy	[137,138]
	2. Palliative: Somatostatin analogs (lanreotide, pasireotide)	[139,142]
**Glucagonoma**	Curative: total pancreatectomy pancreatoduodenectomy with or without splenectomy	[169]
	2. Chemotherapy (streptozicin, fluorouracil, doxorubicin, chlorozotocin, dacarbazin, irinotecan, etoposide, taxanes)	[172]
	3. Somatostatin analogs (octeotride, lanreotide)	[173]

## Data Availability

Not applicable.

## References

[1] Zheng Y., Ley S.H., Hu F.B. (2018). Global aetiology and epidemiology of type 2 diabetes mellitus and its complications. Nat. Rev. Endocrinol..

[2] Resmini E., Minuto F., Colao A., Ferone D. (2009). Secondary diabetes associated with principal endocrinopathies: The impact of new treatment modalities. Acta Diabetol..

[3] American Diabetes Association (2014). Diagnosis and Classification of Diabetes Mellitus. Diabetes Care.

[4] International Expert Committee (2009). International Expert Committee report on the role of the A1C assay in the diagnosis of diabetes. Diabetes Care.

[5] ElSayed N.A., Aleppo G., Aroda V.R., Bannuru R.R., Brown F.M., Bruemmer D., Collins B.S., Gaglia J.L., Hilliard M.E., Isaacs D. (2022). 2. Classification and Diagnosis of Diabetes: Standards of Care in Diabetes—2023. Diabetes Care.

[6] International Diabetes Federation (2021). IDF Diabetes Atlas.

[7] Sherlock M., Ayuk J., Tomlinson J.W., Toogood A.A., Aragon-Alonso A., Sheppard M.C., Bates A.S., Stewart P.M. (2010). Mortality in Patients with Pituitary Disease. Endocr. Rev..

[8] Ershadinia N., Tritos N.A. (2022). Diagnosis and Treatment of Acromegaly: An Update. Mayo Clin. Proc..

[9] Giustina A., Veldhuis J.D. (1998). Pathophysiology of the Neuroregulation of Growth Hormone Secretion in Experimental Animals and the Human. Endocr. Rev..

[10] Clemmons D.R. (2011). Consensus Statement on the Standardization and Evaluation of Growth Hormone and Insulin-like Growth Factor Assays. Clin. Chem..

[11] Caron P., Brue T., Raverot G., Tabarin A., Cailleux A., Delemer B., Renoult P.P., Houchard A., Elaraki F., Chanson P. (2018). Correction to: Signs and symptoms of acromegaly at diagnosis: The physician’s and the patient’s perspectives in the ACRO-POLIS study. Endocrine.

[12] Johannsson G., Bidlingmaier M., Biller B.M.K., Boguszewski M., Casanueva F.F., Chanson P., E Clayton P., Choong C.S., Clemmons D., Dattani M. (2018). Growth Hormone Research Society perspective on biomarkers of GH action in children and adults. Endocr. Connect..

[13] Katznelson L., Laws E.R., Melmed S., Molitch M.E., Murad M.H., Utz A., Wass J.A.H. (2014). Acromegaly: An Endocrine Society Clinical Practice Guideline. J. Clin. Endocrinol. Metab..

[14] Pivonello R., Auriemma R.S., Grasso L.F.S., Pivonello C., Simeoli C., Patalano R., Galdiero M., Colao A. (2017). Complications of acromegaly: Cardiovascular, respiratory and metabolic comorbidities. Pituitary.

[15] Alexopoulou O., Bex M., Kamenicky P., Mvoula A.B., Chanson P., Maiter D. (2013). Prevalence and risk factors of impaired glucose tolerance and diabetes mellitus at diagnosis of acromegaly: A study in 148 patients. Pituitary.

[16] Ciresi A., Amato M.C., Pivonello R., Nazzari E., Grasso L.F., Minuto F., Ferone D., Colao A., Giordano C. (2013). The Metabolic Profile in Active Acromegaly is Gender-Specific. J. Clin. Endocrinol. Metab..

[17] Dal J., List E.O., Jørgensen J.O.L., Berryman D.E. (2015). Glucose and Fat Metabolism in Acromegaly: From Mice Models to Patient Care. Neuroendocrinology.

[18] del Rincon J.-P., Iida K., Gaylinn B.D., McCurdy C.E., Leitner J.W., Barbour L.A., Kopchick J.J., Friedman J.E., Draznin B., Thorner M.O. (2007). Growth Hormone Regulation of p85α Expression and Phosphoinositide 3-Kinase Activity in Adipose Tissue. Diabetes.

[19] Kasayama S., Otsuki M., Takagi M., Saito H., Sumitani S., Kouhara H., Koga M., Saitoh Y., Ohnishi T., Arita N. (2000). Impaired β-cell function in the presence of reduced insulin sensitivity determines glucose tolerance status in acromegalic patients. Clin. Endocrinol..

[20] Kinoshita Y., Fujii H., Takeshita A., Taguchi M., Miyakawa M., Oyama K., Yamada S., Takeuchi Y. (2011). Impaired glucose metabolism in Japanese patients with acromegaly is restored after successful pituitary surgery if pancreatic β-cell function is preserved. Eur. J. Endocrinol..

[21] Frara S., Maffezzoni F., Mazziotti G., Giustina A. (2016). Current and Emerging Aspects of Diabetes Mellitus in Acromegaly. Trends Endocrinol. Metab..

[22] Melmed S. (2020). Pituitary-Tumor Endocrinopathies. N. Engl. J. Med..

[23] Colao A., Auriemma R.S., Galdiero M., Lombardi G., Pivonello R. (2009). Effects of Initial Therapy for Five Years with Somatostatin Analogs for Acromegaly on Growth Hormone and Insulin-Like Growth Factor-I Levels, Tumor Shrinkage, and Cardiovascular Disease: A Prospective Study. J. Clin. Endocrinol. Metab..

[24] Ferraù F., Albani A., Ciresi A., Giordano C., Cannavò S. (2018). Diabetes Secondary to Acromegaly: Physiopathology, Clinical Features and Effects of Treatment. Front. Endocrinol..

[25] Gadelha M.R., Bronstein M.D., Brue T., Coculescu M., Fleseriu M., Guitelman M., Pronin V., Raverot G., Shimon I., Lievre K.K. (2014). Pasireotide versus continued treatment with octreotide or lanreotide in patients with inadequately controlled acromegaly (PAOLA): A randomised, phase 3 trial. Lancet Diabetes Endocrinol..

[26] Hannon A.M., Thompson C.J., Sherlock M. (2017). Diabetes in Patients with Acromegaly. Curr. Diabetes Rep..

[27] Giustina A., Ambrosio M.R., Peccoz P.B., Bogazzi F., Cannavo’ S., De Marinis L., De Menis E., Grottoli S., Pivonello R. (2014). Use of Pegvisomant in acromegaly. An Italian Society of Endocrinology guideline. J. Endocrinol. Investig..

[28] Pijl H., Ohashi S., Matsuda M., Miyazaki Y., Mahankali A., Kumar V., Pipek R., Iozzo P., Lancaster J.L., Cincotta A.H. (2000). Bromocriptine: A novel approach to the treatment of type 2 diabetes. Diabetes Care.

[29] Giustina A., Barkan A., Beckers A., Biermasz N., Biller B.M.K., Boguszewski C., Bolanowski M., Bonert V., Bronstein M.D., Casanueva F.F. (2019). A Consensus on the Diagnosis and Treatment of Acromegaly Comorbidities: An Update. J. Clin. Endocrinol. Metab..

[30] Zaina A., Prencipe N., Golden E., Berton A.M., Arad E., Abid A., Shehadeh J., Kassem S., Ghigo E. (2023). How to position sodium-glucose co-transporter 2 inhibitors in the management of diabetes in acromegaly patients. Endocrine.

[31] Youssef M.E., Yahya G., Popoviciu M.S., Cavalu S., Abd-Eldayem M.A., Saber S. (2023). Unlocking the Full Potential of SGLT2 Inhibitors: Expanding Applications beyond Glycemic Control. Int. J. Mol. Sci..

[32] Chaudhry H.S., Singh G. (2022). Cushing Syndrome. StatPearls [Internet].

[33] Raff H., Carroll T. (2015). Cushing’s syndrome: From physiological principles to diagnosis and clinical care. J. Physiol..

[34] Spiga F., Walker J.J., Terry J.R., Lightman S.L. (2014). HPA Axis-Rhythms. Compr. Physiol..

[35] Jacobson L. (2005). Hypothalamic–Pituitary–Adrenocortical Axis Regulation. Endocrinol. Metab. Clin. N. Am..

[36] Nieman L.K., Biller B.M.K., Findling J.W., Newell-Price J., Savage M.O., Stewart P.M., Montori V.M. (2008). The Diagnosis of Cushing’s Syndrome: An Endocrine Society Clinical Practice Guideline. J. Clin. Endocrinol. Metab..

[37] Badrick E., Kirschbaum C., Kumari M. (2007). The Relationship between Smoking Status and Cortisol Secretion. J. Clin. Endocrinol. Metab..

[38] Wood P.J., Barth J.H., Freedman D.B., Perry L., Sheridan B. (1997). Evidence for the Low Dose Dexamethasone Suppression Test to Screen for Cushing’s Syndrome—Recommendations for a Protocol for Biochemistry Laboratories. Ann. Clin. Biochem. Int. J. Biochem. Lab. Med..

[39] Lacroix A., Feelders R.A., Stratakis C.A., Nieman L.K. (2015). Cushing’s syndrome. Lancet.

[40] Tsagarakis S., Tsigos C., Vasiliou V., Tsiotra P., Kaskarelis J., Sotiropoulou C., Raptis S.A., Thalassinos N. (2002). The Desmopressin and Combined CRH-Desmopressin Tests in the Differential Diagnosis of ACTH-Dependent Cushing’s Syndrome: Constraints Imposed by the Expression of V2 Vasopressin Receptors in Tumors with Ectopic ACTH Secretion. J. Clin. Endocrinol. Metab..

[41] Fleseriu M., Auchus R., Bancos I., Ben-Shlomo A., Bertherat J., Biermasz N.R., Boguszewski C.L., Bronstein M.D., Buchfelder M., Carmichael J.D. (2021). Consensus on diagnosis and management of Cushing’s disease: A guideline update. Lancet Diabetes Endocrinol..

[42] Li D., El Kawkgi O.M., Henriquez A.F., Bancos I. (2020). Cardiovascular risk and mortality in patients with active and treated hypercortisolism. Gland. Surg..

[43] Scaroni C., Zilio M., Foti M., Boscaro M. (2017). Glucose Metabolism Abnormalities in Cushing Syndrome: From Molecular Basis to Clinical Management. Endocr. Rev..

[44] Barbot M., Ceccato F., Scaroni C. (2018). Diabetes Mellitus Secondary to Cushing’s Disease. Front. Endocrinol..

[45] Seckl J.R. (2004). Glucocorticoids and 11beta-Hydroxysteroid Dehydrogenase in Adipose Tissue. Recent Prog. Horm. Res..

[46] Pivonello R., De Leo M., Vitale P., Cozzolino A., Simeoli C., De Martino M.C., Lombardi G., Colao A. (2010). Pathophysiology of Diabetes Mellitus in Cushing’s Syndrome. Neuroendocrinology.

[47] Brennan-Speranza T.C., Henneicke H., Gasparini S.J., Blankenstein K.I., Heinevetter U., Cogger V.C., Svistounov D., Zhang Y., Cooney G.J., Buttgereit F. (2012). Osteoblasts mediate the adverse effects of glucocorticoids on fuel metabolism. J. Clin. Investig..

[48] Nieman L.K., Biller B.M.K., Findling J.W., Murad M.H., Newell-Price J., Savage M.O., Tabarin A. (2015). Treatment of Cushing’s Syndrome: An Endocrine Society Clinical Practice Guideline. J. Clin. Endocrinol. Metab..

[49] Corcuff J.-B., Young J., Masquefa-Giraud P., Chanson P., Baudin E., Tabarin A. (2015). Rapid control of severe neoplastic hypercortisolism with metyrapone and ketoconazole. Eur. J. Endocrinol..

[50] Herndon J., Kaur R.J., Romportl M., Smith E., Koenigs A., Partlow B., Arteaga L., Bancos I. (2021). The Effect of Curative Treatment on Hyperglycemia in Patients with Cushing Syndrome. J. Endocr. Soc..

[51] Suh S., Park M.K. (2017). Glucocorticoid-Induced Diabetes Mellitus: An Important but Overlooked Problem. Endocrinol. Metab..

[52] Popoviciu M.-S., Păduraru L., Yahya G., Metwally K., Cavalu S. (2023). Emerging Role of GLP-1 Agonists in Obesity: A Comprehensive Review of Randomised Controlled Trials. Int. J. Mol. Sci..

[53] Clore J.N., Thurby-Hay L. (2009). Glucocorticoid-Induced Hyperglycemia. Endocr. Pract..

[54] Lansang M.C., Hustak L.K. (2011). Glucocorticoid-induced diabetes and adrenal suppression: How to detect and manage them. Clevel. Clin. J. Med..

[55] Dahia P.L. (2017). Pheochromocytomas and Paragangliomas, Genetically Diverse and Minimalist, All at Once!. Cancer Cell.

[56] Farrugia F.-A., Charalampopoulos A. (2019). Pheochromocytoma. Endocr. Regul..

[57] Tevosian S.G., Ghayee H.K. (2019). Pheochromocytomas and Paragangliomas. Endocrinol. Metab. Clin. N. Am..

[58] Zuber S.M., Kantorovich V., Pacak K. (2011). Hypertension in Pheochromocytoma: Characteristics and Treatment. Endocrinol. Metab. Clin. N. Am..

[59] Manger W.M. (2009). The Protean Manifestations of Pheochromocytoma. Horm. Metab. Res..

[60] Jain A., Baracco R., Kapur G. (2019). Pheochromocytoma and paraganglioma—An update on diagnosis, evaluation, and management. Pediatr. Nephrol..

[61] Lenders J.W.M., Duh Q.-Y., Eisenhofer G., Gimenez-Roqueplo A.-P., Grebe S.K.G., Murad M.H., Naruse M., Pacak K., Young W.F. (2014). Pheochromocytoma and Paraganglioma: An Endocrine Society Clinical Practice Guideline. J. Clin. Endocrinol. Metab..

[62] La Batide-Alanore A., Chatellier G., Plouin P.-F. (2003). Diabetes as a marker of pheochromocytoma in hypertensive patients. J. Hypertens..

[63] Beninato T., Kluijfhout W.P., Drake F.T., Lim J., Kwon J.S., Xiong M., Shen W.T., Gosnell J.E., Liu C., Suh I. (2016). Resection of Pheochromocytoma Improves Diabetes Mellitus in the Majority of Patients. Ann. Surg. Oncol..

[64] Chen Y., Hodin R.A., Pandolfi C., Ruan D.T., McKenzie T.J. (2014). Hypoglycemia after resection of pheochromocytoma. Surgery.

[65] Sherwin R.S., Sacca L. (1984). Effect of epinephrine on glucose metabolism in humans: Contribution of the liver. Am. J. Physiol. Metab..

[66] Smith T.J., Hegedüs L. (2016). Graves’ Disease. N. Engl. J. Med..

[67] McLeod D.S.A., Cooper D.S. (2012). The incidence and prevalence of thyroid autoimmunity. Endocrine.

[68] Davies T.F., Andersen S., Latif R., Nagayama Y., Barbesino G., Brito M., Eckstein A.K., Stagnaro-Green A., Kahaly G.J. (2020). Graves’ disease. Nat. Rev. Dis. Prim..

[69] Bahn R.S. (2010). Graves’ Ophthalmopathy. N. Engl. J. Med..

[70] Wémeau J.-L., Klein M., Sadoul J.-L., Briet C., Vélayoudom-Céphise F.-L. (2018). Graves’ disease: Introduction, epidemiology, endogenous and environmental pathogenic factors. Ann. d’Endocrinologie.

[71] Boelaert K., Torlinska B., Holder R.L., Franklyn J.A. (2010). Older Subjects with Hyperthyroidism Present with a Paucity of Symptoms and Signs: A Large Cross-Sectional Study. J. Clin. Endocrinol. Metab..

[72] Burch H.B., Cooper D.S. (2015). Management of Graves Disease. JAMA.

[73] Díez J.J. (2003). Hyperthyroidism in Patients Older than 55 Years: An Analysis of the Etiology and Management. Gerontology.

[74] Rundle F.F., Wilson C.W. (1945). Development and course of exophthalmos and ophthalmoplegia in Graves’ disease with special reference to the effect of thyroidectomy. Clin. Sci..

[75] Antonelli A., Fallahi P., Elia G., Ragusa F., Paparo S.R., Ruffilli I., Patrizio A., Gonnella D., Giusti C., Virili C. (2020). Graves’ disease: Clinical manifestations, immune pathogenesis (cytokines and chemokines) and therapy. Best Pract. Res. Clin. Endocrinol. Metab..

[76] Drexhage H.A. (2006). Are There More than Antibodies to the Thyroid-Stimulating Hormone Receptor that Meet the Eye in Graves’ Disease?. Endocrinology.

[77] Cappelli C., Pirola I., De Martino E., Agosti B., Delbarba A., Castellano M., Rosei E.A. (2008). The role of imaging in Graves’ disease: A cost-effectiveness analysis. Eur. J. Radiol..

[78] Kahaly G.J., Bartalena L., Hegedüs L., Leenhardt L., Poppe K., Pearce S.H. (2018). 2018 European Thyroid Association Guideline for the Management of Graves’ Hyperthyroidism. Eur. Thyroid. J..

[79] Subekti I., Pramono L.A. (2018). Current Diagnosis and Management of Graves’ Disease. Acta Medica Indones..

[80] Corvilain B., Hamy A., Brunaud L., Borson-Chazot F., Orgiazzi J., Hachmi L.B., Semrouni M., Rodien P., Lussey-Lepoutre C. (2018). Treatment of adult Graves’ disease. Ann. d’Endocrinologie.

[81] Kahaly G.J. (2020). Management of Graves Thyroidal and Extrathyroidal Disease: An Update. J. Clin. Endocrinol. Metab..

[82] Bartalena L., Baldeschi L., Boboridis K., Eckstein A., Kahaly G.J., Marcocci C., Perros P., Salvi M., Wiersinga W.M., on behalf of the European Group on Graves’ Orbitopathy (EUGOGO) (2016). The 2016 European Thyroid Association/European Group on Graves’ Orbitopathy Guidelines for the Management of Graves’ Orbitopathy. Eur. Thyroid. J..

[83] Song E., Koo M.J., Noh E., Hwang S.Y., Park M.J., A Kim J., Roh E., Choi K.M., Baik S.H., Cho G.J. (2021). Risk of Diabetes in Patients with Long-Standing Graves’ Disease: A Longitudinal Study. Endocrinol. Metab..

[84] O’Meara N.M., Blackman J.D., Sturis J., Polonsky K.S. (1993). Alterations in the kinetics of C-peptide and insulin secretion in hyperthyroidism. J. Clin. Endocrinol. Metab..

[85] Bech K., Damsbo P., Eldrup E., Beck-Nielsen H., Røder M.E., Hartling S.G., Vølund A., Madsbad S. (1996). β-Cell function and glucose and lipid oxidation in Graves’ disease. Clin. Endocrinol..

[86] Nishi M. (2018). Diabetes mellitus and thyroid diseases. Diabetol. Int..

[87] Duntas L.H., Orgiazzi J., Brabant G. (2011). The interface between thyroid and diabetes mellitus. Clin. Endocrinol..

[88] Hage M., Zantout M.S., Azar S.T. (2011). Thyroid Disorders and Diabetes Mellitus. J. Thyroid. Res..

[89] Maxon H.R., Kreines K.W., Goldsmith R.E., Knowles H.C. (1975). Long-Term Observations of Glucose Tolerance in Thyrotoxic Patients. Arch. Intern. Med..

[90] Perumal N.L., Selvi J., Sridharan K., Sahoo J., Kamalanathan S. (2019). Insulin Sensitivity and Beta-Cell Function in Graves’ Disease and Their Changes with the Carbimazole-Induced Euthyroid State. Eur. Thyroid. J..

[91] Biondi B., Kahaly G.J., Robertson R.P. (2019). Thyroid Dysfunction and Diabetes Mellitus: Two Closely Associated Disorders. Endocr. Rev..

[92] Anil C., Kut A., Atesagaoglu B., Nar A., Tutuncu N.B., Gursoy A. (2016). Metformin Decreases Thyroid Volume and Nodule Size in Subjects with Insulin Resistance: A Preliminary Study. Med. Princ. Pract..

[93] Funder J.W., Carey R.M., Mantero F., Murad M.H., Reincke M., Shibata H., Stowasser M., Young W.F. (2016). The Management of Primary Aldosteronism: Case Detection, Diagnosis, and Treatment: An Endocrine Society Clinical Practice Guideline. J. Clin. Endocrinol. Metab..

[94] Calhoun D.A., Nishizaka M.K., Zaman M.A., Thakkar R.B., Weissmann P. (2002). Hyperaldosteronism among Black and White Subjects With Resistant Hypertension. Hypertension.

[95] Carey R.M., Siragy H.M. (2003). Newly Recognized Components of the Renin-Angiotensin System: Potential Roles in Cardiovascular and Renal Regulation. Endocr. Rev..

[96] Carey R.M. (2010). Aldosterone and cardiovascular disease. Curr. Opin. Endocrinol. Diabetes.

[97] Carey R.M. (2012). Primary aldosteronism. J. Surg. Oncol..

[98] Mulatero P., Stowasser M., Loh K.-C., Fardella C.E., Gordon R.D., Mosso L., Gomez-Sanchez C.E., Veglio F., Young W.F. (2004). Increased Diagnosis of Primary Aldosteronism, Including Surgically Correctable Forms, in Centers from Five Continents. J. Clin. Endocrinol. Metab..

[99] Calhoun D.A., Jones D., Textor S., Goff D.C., Murphy T.P., Toto R.D., White A., Cushman W.C., White W., Sica D. (2008). Resistant Hypertension: Diagnosis, Evaluation, and Treatment. Hypertension.

[100] Rossi G.-P., Sechi L.A., Giacchetti G., Ronconi V., Strazzullo P., Funder J.W. (2008). Primary aldosteronism: Cardiovascular, renal and metabolic implications. Trends Endocrinol. Metab..

[101] Monticone S., D’Ascenzo F., Moretti C., Williams T.A., Veglio F., Gaita F., Mulatero P. (2018). Cardiovascular events and target organ damage in primary aldosteronism compared with essential hypertension: A systematic review and meta-analysis. Lancet Diabetes Endocrinol..

[102] Sowers J.R., Whaley-Connell D.A., Epstein M. (2009). Narrative Review: The Emerging Clinical Implications of the Role of Aldosterone in the Metabolic Syndrome and Resistant Hypertension. Ann. Intern. Med..

[103] Tuck M.L., Sowers J., Dornfeld L., Kledzik G., Maxwell M. (1981). The Effect of Weight Reduction on Blood Pressure, Plasma Renin Activity, and Plasma Aldosterone Levels in Obese Patients. N. Engl. J. Med..

[104] Gregoire J.R. (1994). Adjustment of the Osmostat in Primary Aldosteronism. Mayo Clin. Proc..

[105] Tomaschitz A., Pilz S. (2010). Aldosterone to Renin Ratio—A Reliable Screening Tool for Primary Aldosteronism?. Horm. Metab. Res..

[106] Lee F.T., Elaraj D. (2019). Evaluation and Management of Primary Hyperaldosteronism. Surg. Clin. N. Am..

[107] Mulatero P., Monticone S., Bertello C., Mengozzi G., Tizzani D., Iannaccone A., Veglio F. (2010). Confirmatory Tests in the Diagnosis of Primary Aldosteronism. Horm. Metab. Res..

[108] Conn J.W., Knopf R.F., Nesbit R.M. (1964). Clinical characteristics of primary aldosteronism from an analysis of 145 cases. Am. J. Surg..

[109] Luther J.M. (2014). Effects of aldosterone on insulin sensitivity and secretion. Steroids.

[110] Stavropoulos K., Imprialos K., Papademetriou V., Faselis C., Tsioufis K., Dimitriadis K., Doumas M. (2020). Primary Aldosteronism: Novel Insights. Curr. Hypertens. Rev..

[111] Young W.F., Stanson A.W., Thompson G.B., Grant C.S., Farley D.R., van Heerden J.A. (2004). Role for adrenal venous sampling in primary aldosteronism. Surgery.

[112] Mulatero P., Bertello C., Rossato D., Mengozzi G., Milan A., Garrone C., Giraudo G., Passarino G., Garabello D., Verhovez A. (2008). Roles of Clinical Criteria, Computed Tomography Scan, and Adrenal Vein Sampling in Differential Diagnosis of Primary Aldosteronism Subtypes. J. Clin. Endocrinol. Metab..

[113] Zennaro M.-C., Boulkroun S., Fernandes-Rosa F.L. (2020). Pathogenesis and treatment of primary aldosteronism. Nat. Rev. Endocrinol..

[114] Steichen O., Zinzindohoué F., Plouin P.-F., Amar L. (2012). Outcomes of Adrenalectomy in Patients with Unilateral Primary Aldosteronism: A Review. Horm. Metab. Res..

[115] Rossi G.P., Cesari M., Cuspidi C., Maiolino G., Cicala M.V., Bisogni V., Mantero F., Pessina A.C. (2013). Long-Term Control of Arterial Hypertension and Regression of Left Ventricular Hypertrophy with Treatment of Primary Aldosteronism. Hypertension.

[116] Wu V.-C., Wang S.-M., Chang C.-H., Hu Y.-H., Lin L.-Y., Lin Y.-H., Chueh S.-C.J., Chen L., Wu K.-D. (2016). Long term outcome of Aldosteronism after target treatments. Sci. Rep..

[117] Lin Y.-H., Lin L.-Y., Chen A., Wu X.-M., Lee J.-K., Su T.-C., Wu V.-C., Chueh S.-C., Lin W.-C., Lo M.-T. (2011). Adrenalectomy improves increased carotid intima-media thickness and arterial stiffness in patients with aldosterone producing adenoma. Atherosclerosis.

[118] Komada H., Hirota Y., So A., Nakamura T., Okuno Y., Fukuoka H., Iguchi G., Takahashi Y., Sakaguchi K., Ogawa W. (2019). Insulin secretion and sensitivity before and after surgical treatment for aldosterone-producing adenoma. Diabetes Metab..

[119] Catena C., Lapenna R., Baroselli S., Nadalini E., Colussi G., Novello M., Favret G., Melis A., Cavarape A., Sechi L.A. (2006). Insulin Sensitivity in Patients with Primary Aldosteronism: A Follow-Up Study. J. Clin. Endocrinol. Metab..

[120] de Herder W.W., Zandee W.T., Hofland J., Feingold K.R., Anawalt B., Blackman M.R., Boyce A., Chrousos G., Corpas E., de Herder W.W., Dhatariya K., Hofland J., Dungan K. (2000). Somatostatinoma. Endotext [Internet].

[121] de Herder W.W., Rehfeld J.F., Kidd M., Modlin I.M. (2015). A short history of neuroendocrine tumours and their peptide hormones. Best Pract. Res. Clin. Endocrinol. Metab..

[122] Rorsman P., Huising M.O. (2018). The somatostatin-secreting pancreatic δ-cell in health and disease. Nat. Rev. Endocrinol..

[123] Kazanjian K.K., Reber H.A., Hines O.J. (2006). Resection of Pancreatic Neuroendocrine Tumors. Arch. Surg..

[124] Cao X.-P., Liu Y.-Y., Xiao H.-P., Li Y.-B., Wang L.-T., Xiao P. (2009). Pancreatic somatostatinoma characterized by extreme hypoglycemia. Chin. Med. J..

[125] Wright J., Abolfathi A., Penman E., Marks V. (1980). Pancreatic Somatostatinoma Presenting with Hypoglycaemia. Clin. Endocrinol..

[126] O’Grady H., Conlon K. (2008). Pancreatic neuroendocrine tumours. Eur. J. Surg. Oncol. (EJSO).

[127] Schubert M.L., Rehfeld J.F. (2019). Gastric Peptides-Gastrin and Somatostatin. Buy Physiol..

[128] Anderson C.W., Bennett J.J. (2016). Clinical Presentation and Diagnosis of Pancreatic Neuroendocrine Tumors. Surg. Oncol. Clin. N. Am..

[129] Elangovan A., Zulfiqar H. (2022). Somatostatinoma. StatPearls [Internet].

[130] Mansour J.C., Chen H. (2004). Pancreatic endocrine tumors. J. Surg. Res..

[131] O’Brien T.D., Chejfec G., Prinz A.R. (1993). Clinical features of duodenal somatostatinomas. Surgery.

[132] Tanaka S., Yamasaki S., Matsushita H., Ozawa Y., Kurosaki A., Takeuchi K., Hoshihara Y., Doi T., Watanabe G., Kawaminami K. (2000). Duodenal somatostatinoma: A case report and review of 31 cases with special reference to the relationship between tumor size and metastasis. Pathol. Int..

[133] Gallo M., Ruggeri R.M., Muscogiuri G., Pizza G., Faggiano A., Colao A. (2018). Diabetes and pancreatic neuroendocrine tumours: Which interplays, if any?. Cancer Treat. Rev..

[134] Mozell E., Woltering E.A., Stenzel P., Rösch J., O’Dorisio T.M. (1990). Functional endocrine tumors of the pancreas: Clinical presentation, diagnosis, and treatment. Curr. Probl. Surg..

[135] Ito T., Igarashi H., Jensen R.T. (2012). Pancreatic neuroendocrine tumors: Clinical features, diagnosis and medical treatment: Advances. Best Pract. Res. Clin. Gastroenterol..

[136] Ramage J.K., Ahmed A., Ardill J., Bax N., Breen D.J., E Caplin M., Corrie P., Davar J., Davies A.H., Lewington V. (2011). Guidelines for the management of gastroenteropancreatic neuroendocrine (including carcinoid) tumours (NETs). Gut.

[137] Kunz P.L., Reidy-Lagunes D., Anthony L.B., Bertino E.M., Brendtro K.B., Chan J.A., Chen H., Jensen R.T., Kim M.K.M., Klimstra D.S. (2013). Consensus Guidelines for the Management and Treatment of Neuroendocrine Tumors. Pancreas.

[138] Williamson J., Thorn C., Spalding D., Williamson R. (2011). Pancreatic and peripancreatic somatostatinomas. Ind. Mark. Manag..

[139] Öberg K., Knigge U., Kwekkeboom D., Perren A., on behalf of the ESMO Guidelines Working Group (2012). Neuroendocrine gastro-entero-pancreatic tumors: ESMO Clinical Practice Guidelines for diagnosis, treatment and follow-up. Ann. Oncol..

[140] Angeletti S., Corleto V.D., Schillaci O., Marignani M., Annibale B., Moretti A., Silecchia G., Scopinaro F., Basso N., Bordi C. (1998). Use of the somatostatin analogue octreotide to localise and manage somatostatin-producing tumours. Gut.

[141] Caplin M.E., Pavel M., Ćwikła J.B., Phan A.T., Raderer M., Sedláčková E., Cadiot G., Wolin E.M., Capdevila J., Wall L. (2014). Lanreotide in Metastatic Enteropancreatic Neuroendocrine Tumors. N. Engl. J. Med..

[142] Al-Toubah T., Schell M.J., Cives M., Zhou J.-M., Soares H.P., Strosberg J.R. (2019). A Phase II Study of Ibrutinib in Advanced Neuroendocrine Neoplasms. Neuroendocrinology.

[143] Ellison T.A., Edil B.H. (2012). The Current Management of Pancreatic Neuroendocrine Tumors. Adv. Surg..

[144] Adeva-Andany M.M., Rañal-Muíño E., Vila-Altesor M., Fernández-Fernández C., Funcasta-Calderón R., Castro-Quintela E. (2019). Dietary habits contribute to define the risk of type 2 diabetes in humans. Clin. Nutr. ESPEN.

[145] Sandru F., Carsote M., Albu S.E., Valea A., Petca A., Dumitrascu M.C. (2020). Glucagonoma: From skin lesions to the neuroendocrine component (Review). Exp. Ther. Med..

[146] Unger R.H. (1971). Glucagon and the Insulin:Glucagon Ratio in Diabetes and Other Catabolic Illnesses. Diabetes.

[147] Reaven G.M., Chen Y.-D.I., Golay A., Swislocki A.L.M., Jaspan J.B. (1987). Documentation of Hyperglucagonemia throughout the Day in Nonobese and Obese Patients with Noninsulin-Dependent Diabetes Mellitus*. J. Clin. Endocrinol. Metab..

[148] Knop F.K., Aaboe K., Vilsbøll T., Vølund A., Holst J.J., Krarup T., Madsbad S. (2011). Impaired incretin effect and fasting hyperglucagonaemia characterizing type 2 diabetic subjects are early signs of dysmetabolism in obesity. Diabetes Obes. Metab..

[149] Adeva-Andany M.M., Funcasta-Calderón R., Fernández-Fernández C., Castro-Quintela E., Carneiro-Freire N. (2018). Metabolic effects of glucagon in humans. J. Clin. Transl. Endocrinol..

[150] Öberg K. (2018). Management of functional neuroendocrine tumors of the pancreas. Gland. Surg..

[151] Cavalu S., Popa A., Bratu I., Borodi G., Maghiar A. (2015). New Evidences of Key Factors Involved in “Silent Stones” Etiopathogenesis and Trace Elements: Microscopic, Spectroscopic, and Biochemical Approach. Biol. Trace Element Res..

[152] Sandhu S., Jialal I. (2022). Glucagonoma Syndrome. StatPearls [Internet].

[153] McGavran M.H., Unger R.H., Recant L., Polk H.C., Kilo C., Levin M.E. (1966). A Glucagon-Secreting Alpha-Cell Carcinoma of the Pancreas. N. Engl. J. Med..

[154] Kindmark H., Sundin A., Granberg D., Dunder K., Skogseid B., Janson E.T., Welin S., Öberg K., Eriksson B. (2007). Endocrine pancreatic tumors with glucagon hypersecretion: A retrospective study of 23 cases during 20 years. Med. Oncol..

[155] Yao J.C., Eisner M.P., Leary C., Dagohoy C., Phan A., Rashid A., Hassan M., Evans D.B. (2007). Population-Based Study of Islet Cell Carcinoma. Ann. Surg. Oncol..

[156] Filho F.d.A.C., Feitosa R.G.F., Fechine C.O.C., de Matos C.M.M., Cardoso A.L., Cardoso D.L. (2015). Glucagonoma syndrome associated with necrolytic migratory erythema. Rev. Assoc. Méd. Bras..

[157] Wermers R.A., Fatourechi V., Wynne A.G., Kvols L.K., Lloyd R.V. (1996). The Glucagonoma Syndrome Clinical and Pathologic Features in 21 Patients. Medicine.

[158] Song X., Zheng S., Yang G., Xiong G., Cao Z., Feng M., Zhang T., Zhao Y. (2017). Glucagonoma and the glucagonoma syndrome (Review). Oncol. Lett..

[159] Lobo I., Carvalho A., Amaral C., Machado S., Carvalho R. (2010). Glucagonoma syndrome and necrolytic migratory erythema. Int. J. Dermatol..

[160] Toberer F., Hartschuh W., Wiedemeyer K. (2019). Glucagonoma-Associated Necrolytic Migratory Erythema: The Broad Spectrum of the Clinical and Histopathological Findings and Clues to the Diagnosis. Am. J. Dermatopathol..

[161] Soga J., Yakuwa Y. (1998). Glucagonomas/diabetico-dermatogenic syndrome (DDS): A statistical evaluation of 407 reported cases. J. Hepato-Biliary-Pancreatic Surg..

[162] Stacpoole P.W. (1981). The Glucagonoma Syndrome: Clinical Features, Diagnosis, and Treatment. Endocr. Rev..

[163] Wu S.-L., Bai J.-G., Xu J., Ma Q.-Y., Wu Z. (2014). Necrolytic migratory erythema as the first manifestation of pancreatic neuroendocrine tumor. World J. Surg. Oncol..

[164] Lv W.-F., Han J.-K., Liu X., Wang S.-C., Pan B., Xu A. (2015). Imaging features of glucagonoma syndrome: A case report and review of the literature. Oncol. Lett..

[165] Kanakis G., Kaltsas G. (2012). Biochemical markers for gastroenteropancreatic neuroendocrine tumours (GEP-NETs). Best Pract. Res. Clin. Gastroenterol..

[166] Partelli S., Bartsch D.K., Capdevila J., Chen J., Knigge U., Niederle B., van Dijkum E.J.N., Pape U.-F., Pascher A., Ramage J. (2017). ENETS Consensus Guidelines for the Standards of Care in Neuroendocrine Tumours: Surgery for Small Intestinal and Pancreatic Neuroendocrine Tumours. Neuroendocrinology.

[167] Kimura W., Tezuka K., Hirai I. (2011). Surgical management of pancreatic neuroendocrine tumors. Surg. Today.

[168] Ferrarese A., Borello A., Gentile V., Bindi M., Ferrara Y., Solej M., Martino V., Nano M. (2014). Meso-pancreatectomy for pancreatic neuroendocrine tumor. Int. J. Surg..

[169] Yao J.C., Hassan M., Phan A., Dagohoy C., Leary C., Mares J.E., Abdalla E.K., Fleming J.B., Vauthey J.N., Rashid A. (2008). One hundred years after “carcinoid”: Epidemiology of and prognostic factors for neuroendocrine tumors in 35,825 cases in the United States. J. Clin. Oncol..

[170] Yalcin S., Oyan B., Bayraktar Y. (2007). Current medical treatment of pancreatic neuroendocrine tumors. Hepato-Gastroenterology.

[171] Kimbara S., Fujiwara Y., Toyoda M., Chayahara N., Imamura Y., Kiyota N., Mukohara T., Fukunaga A., Oka M., Nishigori C. (2014). Rapid improvement of glucagonoma-related necrolytic migratory erythema with octreotide. Clin. J. Gastroenterol..

[172] Eldor R., Glaser B., Fraenkel M., Doviner V., Salmon A., Gross D.J. (2011). Glucagonoma and the glucagonoma syndrome—Cumulative experience with an elusive endocrine tumour. Clin. Endocrinol..

[173] Han X., Wang D., Kuang T., Rong Y., Lou W. (2016). Glucagonoma syndrome: Report of one case. Transl. Gastroenterol. Hepatol..

